# Geochemistry and microbiology of tropical serpentine soils in the Santa Elena Ophiolite, a landscape-biogeographical approach

**DOI:** 10.1186/s12932-022-00079-5

**Published:** 2022-09-27

**Authors:** Agustín F. Solano-Arguedas, Christopher Boothman, Laura Newsome, Richard A. D. Pattrick, Daniel Arguedas-Quesada, Clare H. Robinson, Jonathan R. Lloyd

**Affiliations:** 1 Williamson Research Centre, Department of Earth and Environmental Sciences, School of Natural Sciences, University of Manchester, Manchester, M13 9PL UK; 2 Forest Resources Unit (Reforesta), Engineering Research Institute (INII) and School of Chemistry, Universidad de Costa Rica, Montes de Oca, San José, 11501-2260 Costa Rica; 3 Camborne School of Mines and Environment and Sustainability Institute, University of Exeter, Penryn, Cornwall, TR10 9FE UK; 4 Sociedad Civil Pro Ambiente Verdiazul CR, Playa Junquillal de Santa Cruz, Guanacaste, 50303 Costa Rica

**Keywords:** Biogeochemistry, Geomicrobiology, Mineralogy, Serpentinized peridotite, Nickel and cobalt laterite, Iron and manganese cycling, Prokaryote, Fungi, Costa Rica, Tropical dry forest

## Abstract

The Santa Elena Ophiolite is a well-studied ultramafic system in Costa Rica mainly comprised of peridotites. Here, tropical climatic conditions promote active laterite formation processes, but the biogeochemistry of the resulting serpentine soils is still poorly understood. The aim of this study was to characterize the soil geochemical composition and microbial community of contrasting landscapes in the area, as the foundation to start exploring the biogeochemistry of metals occurring there. The soils were confirmed as Ni-rich serpentine soils but differed depending on their geographical location within the ophiolite area, showing three serpentine soil types. Weathering processes resulted in *mountain* soils rich in trace metals such as cobalt, manganese and nickel. The lowlands showed geochemical variations despite sharing similar landscapes: the *inner ophiolite lowland* soils were more like the surrounding mountain soils rather than the *north lowland* soils at the border of the ophiolite area, and within the same riparian basin, concentrations of trace metals were higher downstream towards the mangrove area. Microbial community composition reflected the differences in geochemical composition of soils and revealed potential geomicrobiological inputs to local metal biogeochemistry: iron redox cycling bacteria were more abundant in the mountain soils, while more manganese-oxidizing bacteria were found in the lowlands, with the highest relative abundance in the mangrove areas. The fundamental ecological associations recorded in the serpentine soils of the Santa Elena Peninsula, and its potential as a serpentinization endemism hotspot, demonstrate that is a model site to study the biogeochemistry, geomicrobiology and ecology of tropical serpentine areas.

**Graphical Abstract:**

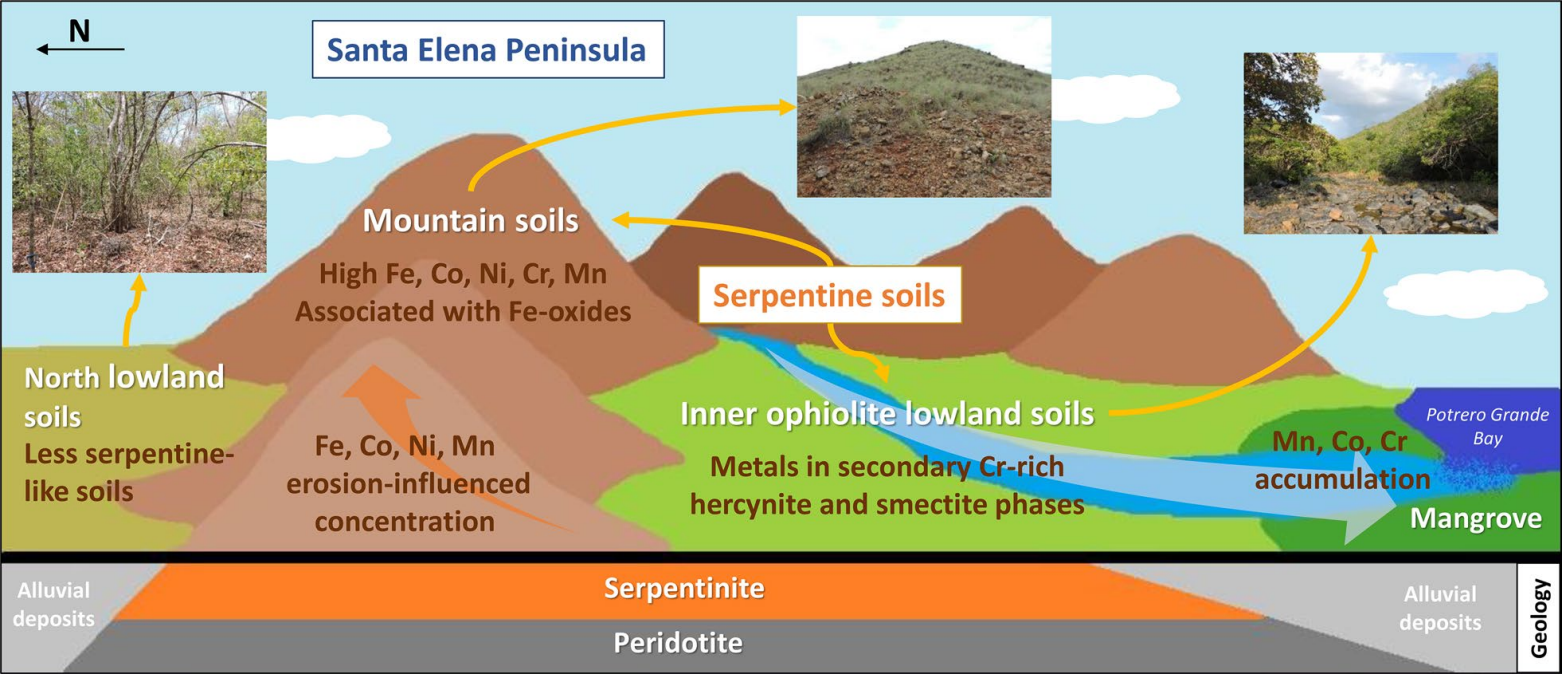

**Supplementary Information:**

The online version contains supplementary material available at 10.1186/s12932-022-00079-5.

## Introduction

The Santa Elena Peninsula (SEP) is on the northwestern Pacific coast of Costa Rica within the Guanacaste Conservation Area (ACG, in Spanish: Área de Conservación Guanacaste) and has a rugged topography including several mountain ranges (elevation > 700 m) and small riparian basins. The majority of the peninsula is composed of the Santa Elena Ophiolite (SEO), one of the oceanic complexes found along the Pacific coast of Costa Rica, formed as a result of accretionary processes during the Upper Cretaceous [1, 2]. The SEO is composed mainly of ultramafic serpentinized peridotites (lherzolites, harzburgites and dunites) in association with mafic lithologies such as gabbros, diabases and basalts [1–4].

In the tropical and sub-tropical zones, the weathering of ultramafic rocks exposed to annual precipitation of over 1000 mm and seasonal temperatures ranges between 15 and 33 °C, leads to development of nickeliferous laterites [5, 6]. Such climatic conditions prevail in the SEP, a tropical region mostly dry to sub-humid where the average annual temperature during the day is 33 °C and 22 °C at night. The average annual precipitation is 1528 mm, with a marked dry–wet seasonality where only 5% of the precipitation occurs during the dry season (December-mid May) [7–9]. Thus, the SEP is an active area of lateritic soil formation.

The dominant soil type covering the SEO is defined as a Lithic Ustorthent, an Entisol that have an ustic soil moisture regime and a lithic contact within 50 cm of the mineral soil surface according to Soil Taxonomy [10], and its distribution on the SEP closely corresponds to the area underlain by the serpentinized peridotite (Additional file 1: Fig. S1a, b) [11, 12]. In Entisols, the parent rock is the predominating soil-forming factor rather than other pedogenic processes stimulated by vegetation or climate [13], and although the soil in Santa Elena has a thin regolith layer, it can be characterized as a laterite due to its oxidized and clayey mineralogy and its water retention capacity [10, 14]. However, the resulting soils can be described as serpentine soils too because their nature and geochemical composition will be determined by the geochemistry of the ultramafic rock from where they were formed and its complex geologic history.[15]. Thus, the particular biogeochemistry of serpentine soils will define the ecology of the associated biological communities, resulting in unique areas with serpentine ecosystems in the SEP [16]. Additionally, recently developed fluvial and alluvial deposits (Quaternary) are found associated with the Potrero Grande basin and the rivers on the south-center of the SEP towards the Potrero Grande bay, and on the northern margin of the ophiolite (Additional file 1: Fig. S1b), resulting in Inceptisols with ustic soil moisture regime of alluvial and colluvial origin sourced from the surrounding mountains [2, 10, 11].

**Fig. 1:**
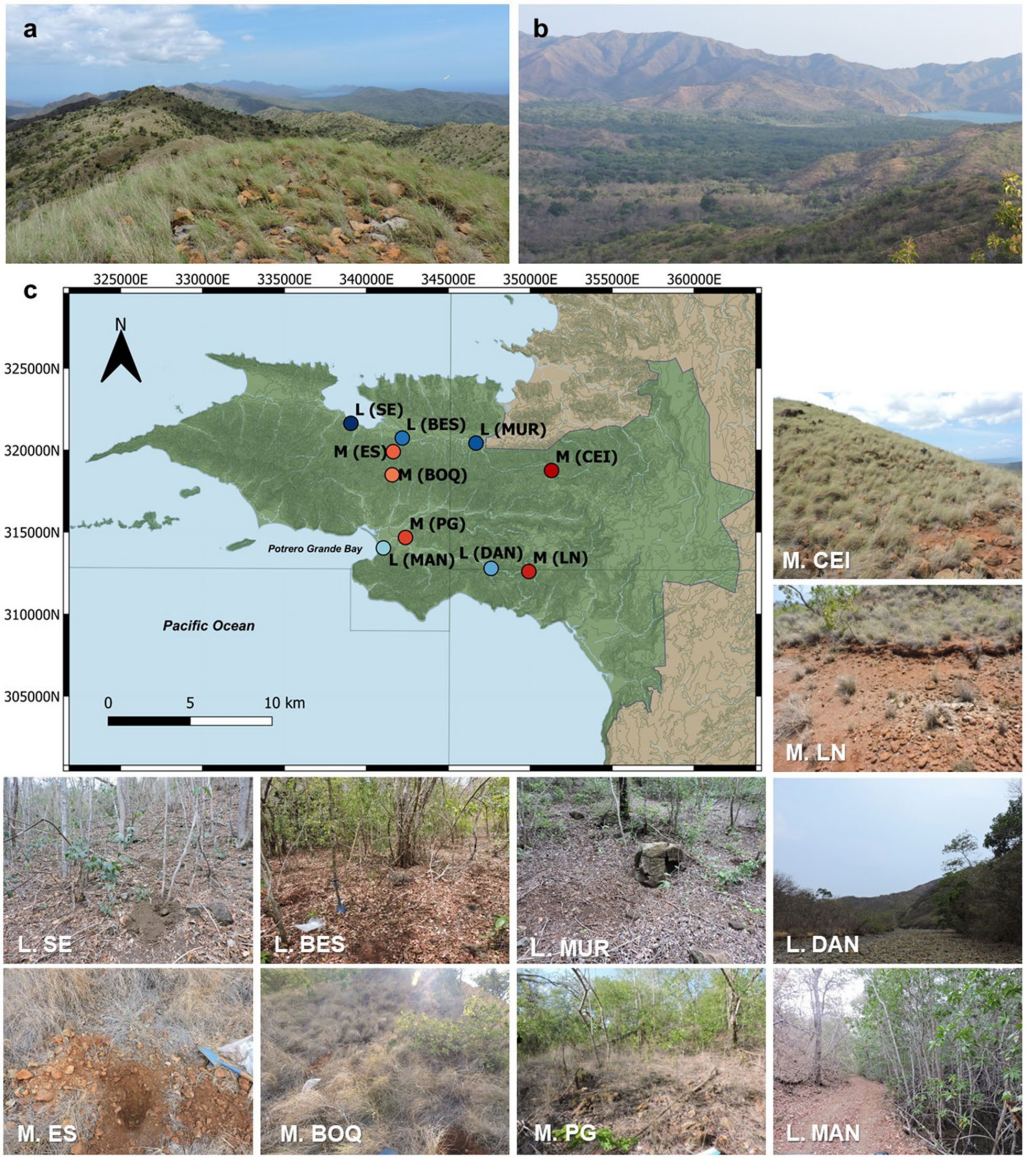
Characteristic landscapes in the SEP. In the mountains such as Cerro el Inglés (**a**), the hilltops and the upper sections of the hills show a semi-deciduous/deciduous forest with scarce and low-stature trees and dominated (foreground) by grasses (Poaceae) with areas of exposed soils. In the lowlands such as in Potrero Grande basin (**b**), the deciduous forest is denser and changes to an evergreen forest in some areas; the mountain landscape can be seen in the foreground and background. **c** Geographical distribution of the 10 locations sampled within the SEP (green area: National Park Santa Rosa). Mountain (M) landscapes are in red-to-orange colors and lowland (L) landscapes in light-to-dark blue (see labels and details in Table 1)

The predominant vegetation macrotype along the SEP is a semi-deciduous/deciduous forest, with its distribution closely related to the area of the SEO (Additional file 1: Fig. S1c). However, the composition changes with the topography of the peninsula; it is dominated by xerophytic shrubs and herbaceous vegetation in the exposed areas or the mountain tops, while a semi-deciduous/deciduous and/or evergreen vegetation is present in the lower altitudes or near water courses. In the alluvia of the Potrero Grande river and across other rivers in the north of the peninsula, the vegetation changes completely to a seasonal evergreen lowland forest, with mangrove forest in some areas like Potrero Grande intertidal zone [7, 17]. The resulting landscape in the SEP overlying the ophiolite area can thus be separated into a predominant *mountain* landscape with scarce shrubby vegetation, closely related to the serpentine soils (Fig. 1a), and the *lowland* landscape with more complex vegetation and influenced by the serpentine soils via alluvial deposits (Fig. 1b).

Serpentine soils have higher concentrations of metals than their parental rocks as a result of weathering processes, including cobalt and nickel [5]. This is of particular interest within a geomicrobiological context, because despite the role of microorganisms has been deeply studied and characterized in the biogeochemical cycles of elements such as iron, manganese, phosphorus, nitrogen or sulfur; it is still widely unexplored for other elements like cobalt and nickel [18–22], and understanding the cycling of these metals within natural systems is an urgent need due to an expanding demand for critical metals [23, 24]. However, the indigenous microorganisms in serpentine soils and their natural biogeochemical implications are yet to be defined, and questions like how these metals accumulate and move in nature are important to improve (bio)extraction strategies and also to address their fate when contamination is a problem. For example, nickel laterites are source of 40% of global Ni production and 20–30% of Co [5, 6, 24], and can be amenable to bioprocessing of both metals, as either an alternative method of extraction from low-grade deposits or to re-process waste materials from mining operations [25]. Bioextraction has also been examined using model acidophilic Fe(III)-reducing bacteria or fungi such as *Aspergillus* sp. and *Penicillium* sp. [26–29].

The SEP is protected as a National Park within an area declared as a World Heritage Site by UNESCO due to its significance in developing ecological and biological processes, and where ore exploitation is prohibited[30]. However, reports of Co, Ni and other metals associated with the laterites of Santa Elena are very limited and focus on geologic features of the ultramafic source rocks, with works on soils or sediments restricted to a small area of the peninsula [1, 2, 31–33]. In addition, work on Santa Elena microorganisms, has been restricted to springs and not related to the soil geochemistry or related biogeochemical processes [34, 35]. This makes the SEP a unique area to study serpentine soils and to develop an understanding of the linkages between the geochemical processes and the associated microbial communities, and how they impact on the biogeochemistry of key metals. This fundamental work could also help inform future bioprocessing work at other sites, with the SEP representing a model location for serpentine and laterites soils.

The aim of this study was to characterize the geochemistry of the serpentine soils in the SEP and the associated microbial communities, as a basis for understanding the biogeochemistry of metals within these soils. We considered a biogeographical approach given the two contrasting landscapes present, mountains and lowlands, as we hypothesize that the differences between landscapes are likely to be the result of variations in soil geochemistry as seen in other serpentine ecosystems. To address this hypothesis, we first described the mineralogy and the geochemical composition of the soils from ten different locations, considering main landscapes present within the area of the SEO. For a better interpretation of the lateritic soil system overall, the geochemistry of serpentinite rocks prior to regolith formation was also determined. Finally, we conducted microbial community analyses, and examined the coupling of our data sets to understand the controlling impacts of geomicrobiology (prokaryotic and fungal communities) and related biogeochemistry on the metals locally present. To our knowledge this is the first microbial-geochemical study on the ecological important serpentine soils associated with the SEO, and, we extend our work to consider our data in the context of different landscapes, soil types, topographies, and the geographical location within the ophiolite area.

## Methods

### Sampling of soils and rocks

Samples were collected in the Santa Elena Peninsula, Costa Rica, within the National Park Santa Rosa of the ACG, during the dry seasons of 2016 (May) and 2017 (April). Ten locations along the peninsula were chosen from the area of the Santa Elena Ophiolite (Additional file 1: Fig. S1). Five sites from mountain landscapes and five from lowlands were sampled, considering representative locations of possible ecological subdivisions present in each landscape type (e.g. topographical gradients of altitude, closeness to rivers or mangroves, the boundaries of the ophiolite area, forest composition, etc.) (Fig. 1c, Table 1). In each site, a sampling area of 5 × 5 m was traced wherein 3 randomly distributed (replicate) sections were taken at a depth of 10–15 cm, pointing to a depth where the influence of both, superficial inputs (vegetation and climate) and underlying bedrock, was plausible (lithic contact was less than 40 cm depth in average). For each sample, the soil from the surface was carefully removed, intending to decrease the rate of organic matter microbiota-decomposers to focus on potential microorganisms more related to mineral soil biogeochemistry [36, 37]. When the depth was achieved, 1–1.5 kg of soil at the same depth was extracted and stored into a re-sealable plastic bag at 4 °C. Large rock fragments (> 5 cm) or large roots were separated from the sample. Serpentinite rocks found loose at the same depth within the soil sampling areas were also collected, representing weathered protolith. Additionally, relatively unweathered rocks were collected from outcrops as close to the sampling locations as possible.

**Table 1:** Summary of the locations sampled within the SEP and their main characteristics

Location	Coordinates	Altitude	Vegetation^a^	Soil type^b^	Geology^c^	Topography
Mountain (CEI)	N10° 53.198′ W85° 41.490′	495 m	Grass only but scarce, with patches of exposed soils	Entisol (Lithic ustorthent)	Ophiolite	Top of a mountain (Cerro el Inglés)
Mountain (LN)	N10° 49.849′ W85° 42.236′	309 m	Grass only but scarce, with exposed soils	Entisol (Lithic ustorthent)	Ophiolite	Top of a mountain (Loma Nance), small terrace
Mountain (PG)	N10° 50.957′ W85° 46.372′	17 m	Semi-deciduous/deciduous forest mixed with grass	Entisol (Lithic ustorthent)	Ophiolite	Lower side of a hill, near Potrero Grande river
Mountain (ES)	N10° 53.797′ W85° 46.796′	197 m	Grass dominated, Semi-deciduous/deciduous forest	Entisol (Lithic ustorthent)	Ophiolite	Top of a mountain (El Silencio road)
Mountain (BOQ)	N10° 53.030′ W85° 46.826′	269 m	Grass dominated, Semi-deciduous/deciduous forest	Entisol (Lithic ustorthent)	Ophiolite	Top of a mountain (Loma Boquerones)
Lowland (DAN)	N10° 49.947′ W85° 43.499′	69 m	Semi-deciduous/deciduous forest, no grass present	Entisol (Lithic ustorthent)	Ophiolite	River terrace surrounded by steep mountains, ~ 10 km upstream from MAN location
Lowland (MAN)	N10° 50.606′ W85° 47.110′	− 4 m	Seasonal evergreen forest of lowlands, no grass present	Inceptisol (Fluventic ustropept)^d^	Sedimentary deposits	Flat area between the base of a hill and the Potrero Grande mangrove flooded area
Lowland (BES)	N10° 54.249′ W85° 46.493′	18 m	Seasonal evergreen forest of lowlands no grass present	Inceptisol (Ustic dystropept)^d^	Ophiolite (north margin)	Flat area on a river valley, close to a river, base of the ES mountains
Lowland (MUR)	N10° 54.094′ W85° 44.025′	52 m	Seasonal evergreen forest of lowlands no grass present	Inceptisol (Ustic dystropept)^d^	Ophiolite (north margin)	Small terrace, close to a creek in Murciélago sector, lower side of a small hill
Lowland (SE)	N10° 54.730′ W85° 48.217′	25 m	Seasonal evergreen forest of lowlands, no grass present	Inceptisol (Fluventic ustropept)^d^	Sedimentary deposits/ Ophiolite (north margin)	Lower side of a hill, on a very steep area

^a^Vegetation, ^b^ soil type and ^c^ geology classifications are based on vegetation, soil taxonomy, and geology maps of ACG [11, 12, 38]. ^d^ These soil types, former suborder Tropepts, are no longer in use in Soil Taxonomy since 1999, and should be replaced by soils of current suborder Ustepts (Inceptisols that have ustic soil moisture regime) [10, 39, 40] (For details of the sampling points in each map see Additional file 1: Fig. S1)

### Geochemical characterization

Prior to bulk geochemical characterization, rock samples were ground into fine powder using a Tema® mill (tungsten). Soil samples were dried at 105 °C for 24 h prior to grinding. Soil water content was calculated as a percentage of the mass lost during drying. The pH of the soils was measured by adding 10 g of fresh sample to 10 mL of deionized water, then measuring after 1 h [41].

#### X-ray diffraction spectroscopy (XRD)

For both rock powder and soil samples, measurements were carried out on a Bruker D8 Advance diffractometer, equipped with a Göbel Mirror, a Lynxeye detector and a copper X-ray tube, providing CuK_α1_ X-rays with a wavelength of 1.5406 Å. Samples were scanned from 5 to 70° 2θ, with a step size of 0.02° and a count time of 0.2 s per step. The resulting patterns were evaluated using EVA version 4 software, comparing experimental data to ICDD (International Centre for Diffraction Data) Database.

#### X-ray fluorescence spectroscopy (XRF)

For both rock powder and soil samples, measurements were carried out on an Axios Panalytical XRF spectrometer equipped with a Rh X-ray source. Samples were scanned, identifying both major (*Omnian program*) and trace elements (*Pro-Trace program*). Sample preparation involved 12 g of dry sample and 3 g of Hoechst Wax™ ground together in an agate ball mill and then pressed into a pellet with a pneumatic press. The total carbon content in the samples was also calculated by loss on ignition (LOI), weighing exactly 1.000 g of dry sample and determining the mass after heating first at 105 °C for 1 h and then at 1000 °C for 1 h.

#### Petrographic examination, and electron probe micro analysis (EPMA)

Polished thin sections (30 μm) of rock samples were prepared using standard techniques. Petrographic analysis was undertaken using a Nikon Eclipse LV100NPOL petrographic microscope equipped with a Nikon DS-Fi2 camera and DS-U3 camera control software. Mineral and textural analysis was undertaken and phases suitable for EPMA identified. The polished thin sections were carbon coated prior to EPMA. EPMA was carried out using a Cameca SX100 electron probe microanalyzer equipped with 5 wavelength dispersive spectrometers (WDS). Elemental distribution maps were created for Fe, Mg, Si, Al, Cr, Mn, Co, Ni, Ca, Ti, Cl, K, Na and S, and points were analyzed at 20 kV, 5 nA with a 10 μm beam using the standards fayalite (Fe), periclase (Mg), anorthite (Si, Al), Cr_2_O_3_, tephrolite (Mn), Co-metal, NiO, wollastonite (Ca), rutile (Ti), sodalite (Cl), orthoclase (K), jadeite (Na) and pyrite (S).

#### Mapping and statistical analyses

The processing of map data was done using QGIS 3.10.0 A Coruña software [42]. GIS layers were taken from the ACG GIS-maps database [43] and Costa Rica Lambert Norte was used as the coordinate reference system. Statistical analyses on XRF data sets were developed with JMP 15.1.0 statistical software [44]. For ANOVA, Wilcoxon tests and for correlations, α = 0.05 was considered appropriate. Hierarchical cluster analysis (HC) and principal component analysis (PCA) were also performed.

### Microbial community analysis

The raw data obtained in this research were deposited to NCBI SRA (Sequence Read Archive; http://www.ncbi.nlm.nih.gov/sra/) under the project accession number: PRJNA606410.

#### DNA extraction

One replicate core from every location was chosen, and DNA was extracted from 200 µl of sediment slurry using a DNeasy PowerLyzer PowerSoil Kit (Qiagen, Manchester, UK). The 16S rRNA amplicon was amplified via PCR (polymerase chain reaction) using 8F (5′-AGAGTTTGATCCTGGCTCAG-3′), and 1492R (5′-TACGGYTACCTTGTTACGACTT-3′) [45]. Following amplification via PCR, the DNA was stained with SYBR Safe DNA Gel Stain (Thermo Fisher Scientific) before placement in an agarose gel, where it was subsequently separated using electrophoresis. The stained DNA was viewed under UV light, and target ~ 1500 base pair products were identified by comparison to a ladder of DNA fragments of varying lengths.

#### Prokaryotic community analysis

The PCR amplicons of the 16S rRNA gene were sequenced using the Illumina MiSeq platform (Illumina, San Diego, CA, USA) targeting the V4 hyper variable region (forward primer, 515F, 5′-GTGYCAGCMGCCGCGGTAA-3′; reverse primer, 806R, 5′-GGACTACHVGGGTWTCTAAT-3′) for 2 × 250-bp paired-end sequencing (Illumina) [46, 47]. PCR amplification was performed using Roche FastStart High Fidelity PCR System (Roche Diagnostics Ltd, Burgess Hill, UK) in 50 μl reactions under the following conditions: initial denaturation at 95 °C for 2 min, followed by 36 cycles of 95 °C for 30 s, 55 °C for 30 s, 72 °C for 1 min, and a final extension step of 5 min at 72 °C. The PCR products were purified and normalized to ~ 20 ng each using the SequalPrep Normalization Kit (Fisher Scientific, Loughborough, UK). The PCR amplicons from all samples were pooled in equimolar ratios. The run was performed using a 4 pM sample library spiked with 4 pM PhiX to a final concentration of 10% following the method of Schloss and Kozich [48].

Raw sequences for prokaryotes were divided into samples by barcodes (up to one mismatch was permitted) using a sequencing pipeline. Quality control and trimming was performed using Cutadapt [49], FastQC [50], and Sickle [51]. MiSeq error correction was performed using SPADes [52]. Forward and reverse reads were incorporated into full-length sequences with Pandaseq [53]. Chimeras were removed using ChimeraSlayer [54], and operational taxonomic units (OTUs) were generated with UPARSE [55]. OTUs were classified by Usearch [56] at the 97% similarity level, and singletons were removed. Rarefaction analysis was conducted using the original detected OTUs in Qiime [57]. The taxonomic assignment was performed by the RDP classifier [58].

#### Fungal community analysis

Sequencing of PCR amplicons of the ITS2 region of nuclear ribosomal DNA was conducted with the Illumina MiSeq platform (Illumina, San Diego, CA, USA), targeting the ITS2 internal transcribed spacer region between the large subunit (LSU) and the 5.8S ribosomal genes (forward primer, ITS4F, 5′-AGCCTCCGCTTATTGATATGCTTAART-3′, reverse primer, 5.8SR, 5′-AACTTTYRRCAAYGGATCWCT-3′;) [59] for 2 × 300-bp paired-end sequencing (Illumina) [46, 47]. PCR amplification was performed using Roche FastStart High Fidelity PCR System (Roche Diagnostics Ltd, Burgess Hill, UK) in 50 μl reactions under the following conditions: initial denaturation at 95 °C for 2 min, followed by 36 cycles of 95 °C for 30 s, 56 °C for 45 s, 72 °C for 2 min, and a final extension step of 5 min at 72 °C. The PCR products were purified and normalized to ~ 20 ng each using the SequalPrep Normalization Kit (Fisher Scientific, Loughborough, UK). The PCR amplicons from all samples were pooled in equimolar ratios. The run was performed using a 10 pM sample library spiked with 10 pM PhiX to a final concentration of 10% following the method of Schloss and Kozich [48].

The ITS sequencing data produced by the Miseq platform was analysed using the PIPITS automated pipeline [60]. Chimeras were removed by reference based chimera detection using UCHIME [61] in conjunction with the UNITE UCHIME reference data set. The taxonomic assignment was performed by the RDP classifier [58] using the UNITE fungal ITS reference data set.

## Results and discussion

### Geochemical characterization of rocks

Before exploring the geochemistry of the serpentine soils of the SEP and their possible biogeochemical implications, serpentinite clasts within the regolith at 10 cm depth were analyzed to determine the geochemistry and mineral composition of the parent rock of these mineral soils. Bulk geochemical composition of all the rock samples were determined by XRF (Additional file 1: Fig. S2). Co showed a strong positive correlation with Mn (r = 0.8765, p < 0.0001) and Ni (r = 0.8530, p < 0.0001), to a lesser degree to Fe (r = 0.5959, p = 0.0071), and Cr (r = 0.4655, p = 0.0438). Mn was correlated to Fe (r = 0.5112, p = 0.0253) similarly than Co, while Cr and Ni showed a closer association with Fe (r = 0.8428, p < 0.0001; r = 0.7790, p < 0.0001, respectively). Cr was also positively correlated with Al (r = 0.7139, p = 0.0006) (Table S1, Additional file 1: Fig. S2).

XRD (Additional file 1: Fig. S3) revealed the bulk mineralogy of these rocks to mainly comprise the serpentine-group mineral lizardite [Mg_3_Si_2_O_5_(OH)_4_] irrespective of geomorphological location and soil type. Despite the high degree of serpentinization, peridotite minerals, notably forsteritic olivine [(Mg,Fe)Si_2_O_4_] and enstatite [MgSiO_3_] were present in different amounts in the bulk mineralogy; the clinopyroxenes augite [(Ca,Na)(Mg,Fe,Al,Ti)(Si,Al)_2_O_6_] and diopside [(Ca,Mg,Fe)SiO_3_] were also present to a lesser extent. This confirmed the peridotitic nature of the parent rock and the presence of extensive lizardite and olivine, along with enstatite as the predominant pyroxene, suggest a harzburgite protolith. Clinochlore [(Mg,Fe)_5_Al_2_Si_3_O_10_(OH)_8_] was also present in minor proportions. Rocks collected from superficial (transported) deposits close to the sampling locations revealed the same mineral suite but with a greater concentration of forsterite in some cases and the absence of clinochlore.

**Fig. 2:**
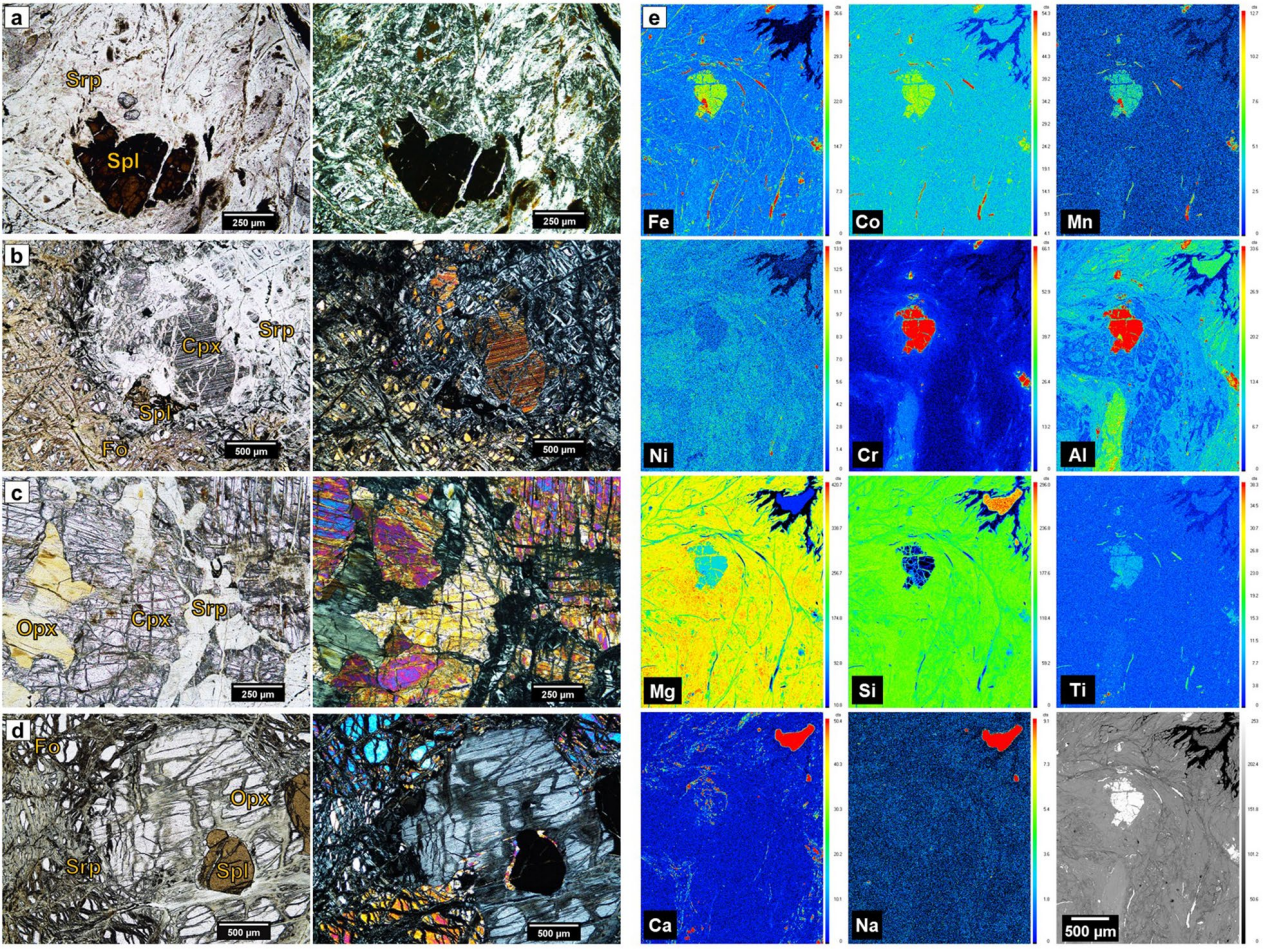
Photomicrographs from polished thin sections of representative serpentinite rocks from SEP. Rock clasts from within the soil collected at 10 cm depth from the Mountain LN (**a**) and Lowland MAN (**b**) sites, rocks from outcrops close to Mountain CEI (**c**) and Lowland BES (**d**) site; under plane-polarized light (left) and crossed polars (right). **e** Elemental distribution determined on a polished thin section of a serpentinite clast from 10 cm depth (**a**), defined by EPMA (rotated 90° counterclockwise). Count intensity color scale (right of each micrograph) decreases downwards. Elemental analysis of **b**, **c** and **d** are shown in Additional file 1: Figs. S4, S5 and S6, respectively. Cpx: clinopyroxene, Fo: forsterite, Ol: olivine, Opx: orthopyroxene, Srp: serpentine, Spl: spinel

The petrographic data confirmed a very high degree of serpentinization of the rocks from mountain locations (Fig. 2a) with pervasive lizardite surrounding relict ferromagnesian minerals; the resistate mineral, a ferrite spinel was also identified, likely to be a ferrite chrome spinel [(Mg,Fe)(Al*,*Cr)_2_O_4_] due to the positive Fe:Cr correlation (Additional file 1: Fig. S2). The in situ physical and chemical weathering of the parent peridotite to produce the mountain soils was found to be more intense than that observed in the lowland soils. In the latter, a higher proportion of relict olivine and pyroxene was observed although they were still dominated by serpentine minerals (lizardite) (Fig. 2b) suggesting that the rock clasts in these lowland colluvial and alluvial deposits are likely to have been brought in by erosion and have had less exposure to the more intense weathering processes in the mountain areas. In rock samples collected from outcrops close to the soil sampling locations, the proportion of the peridotite parent minerals (olivine and pyroxene) to serpentine minerals was much greater than in the soil-hosted clasts (Fig. 2c, d).

The elemental distribution maps (Fig. 2e, Additional file 1: Figs. S4-S6) produced from the polished thin sections of the serpentinite clasts from the soils (Fig. 2a, b) and of the serpentinized peridotites from outcrops (Fig. 2c, d) supported correlations observed in the bulk geochemistry and mineralogy. Olivine was confirmed as forsterite (MgO: 48.8 ± 0.3%, FeO: 9.5 ± 0.1%, SiO_2_: 40.3 ± 0.1%) regardless of rock type (Additional file 1: Figs. S4-S6), and lizardite covered a major proportion of Mg and Si in the serpentinite clasts (Fig. 2e and Additional file 1: Fig. S4) and in the peridotites from outcrops to a lesser extent. Fe was ubiquitous in all minerals although concentrated in Fe oxide veinlets, and in the spinel minerals. Cr, and especially Al were co-located in the spinel with Fe and Mg, confirming the identification of this phase as a hercynite [(Fe,Mg)(Al,Cr)_2_O_4._], also reflected in the positive correlations in the bulk geochemistry (Additional file 1: Fig. S2), but with differences between locations. In the serpentinite clast of the mountain location LN (Fig. 2a) was a Cr-rich hercynite (MgO: 12.8 ± 1.4%, FeO: 19.6 ± 0.9%, Al_2_O_3_: 30.1 ± 4.8%, Cr_2_O_3_: 33.9 ± 5.2%) (Fig. 2e), but in the serpentinite clast from the lowland MAN (Fig. 2b) was an Al-rich hercynite (Additional file 1: Fig. S4), same as in the peridotites from outcrops (MgO: 15.5 ± 0.1%, FeO: 11.5 ± 0.1%, Al_2_O_3_: 53.9 ± 0.3%, Cr_2_O_3_: 15.5 ± 0.2%) (Fig. 2d, Additional file 1: Fig. S6).

Co was very strongly associated with Fe but mostly restricted to Fe-oxide veinlets and spinel minerals, with concentrations ranging from 0.016 wt% to 0.044 wt% in the spinels and slightly higher in the Fe-veinlets (Fig. 2e, S4–S6); Mn behaved similarly with Fe, although Mn was present in all minerals. In the spinel minerals Mn was more concentrated in the Cr-rich hercynite of the serpentinite clast from the mountain LN (0.245 ± 0.030 wt%) (Fig. 2e) than in the Al-rich hercynite of the lowland serpentinite and of the peridotites from outcrops (0.126 ± 0.016 wt%) (Additional file 1: Figs. S4–S6). The observed patterns of Co and Mn linked to Fe were also supported by their positive bulk elemental correlations (Additional file 1: Fig. S2). Ni was associated with serpentine and olivine with concentrations ranging from 0.2 wt % to 0.3 wt % and was also present in spinels in similar concentration. However, in the highly weathered serpentinite clasts (e.g. Mountain LN), Ni was concentrated in Fe oxide veinlets and the lower Al regions of the lizardite rather than in the spinels (Fig. 2e), which can be related to the Ni-spinel/olivine partitioning in the parent peridotite. During lherzolite formation, such as those from the SEO, Ni^2+^ is stabilized in olivines and Cr^3+^ in spinels, due to their high octahedral coordination site preferences [62]. Additionally, in Mg-rich spinel peridotite xenoliths that have higher concentrations of Ni in the olivine than in the spinel or pyroxenes, and more Co in the spinel than the olivine or pyroxenes, the Ni partitioning is ruled by the major Al/Cr composition of the spinel [63]. In the serpentinite clasts of SEP both Al and Cr were strongly associated in the Cr-rich hercynite (Fig. 2e and Additional file 1: Fig. S2, Table S1) explaining the absence of Ni in the hercynite spinels and its presence in the lizardite, the serpentinized product of parental olivines. Finally, clinopyroxenes were confirmed as diopside, as in the lowland MAN serpentinite clast (MgO: 16.1 ± 0.4%, CaO: 21.3 ± 0.5%, Al_2_O_3_: 6.4 ± 0.5%, FeO: 2.8 ± 0.1%, SiO_2_: 50.8 ± 0.4%) (Fig. 2b and Additional file 1: Fig. S4), and orthopyroxenes as enstatite, as in the peridodite from the outcrop close to BES (MgO: 31.5 ± 0.5%, CaO: 1.8 ± 0.6%, Al_2_O_3_: 5.5 ± 0.3%, FeO: 6.3 ± 0.2%, SiO_2_: 53.3 ± 3.5%) (Fig. 2d and Additional file 1: Fig. S6). Co and Ni concentrations in pyroxenes were considerably lower than in the other minerals present (Additional file 1: Fig. S4–S6).

In summary, the rock clasts found within the serpentine soils of the SEP were highly serpentinized peridotites regardless of the landscape or the location. However, relicts of the peridotite parent minerals were more common in rocks found in the rapidly transported lowland soils; rocks in the mountain sites showed a more advanced degree of serpentinization related to the more intense in situ degree of physical and chemical weathering. The geochemical analysis of these rocks, also including those from the outcrops, displayed positive correlations between Fe, Co, Ni, Mn and Cr. Co and Mn were concentrated in Cr-rich spinels and Fe-oxide veinlets. Ni was also present in those minerals but in highly serpentinized rocks it was concentrated in the Fe-veinlets and in the serpentine minerals.

### Geochemical characterization of the serpentine soils

**Fig. 3:**
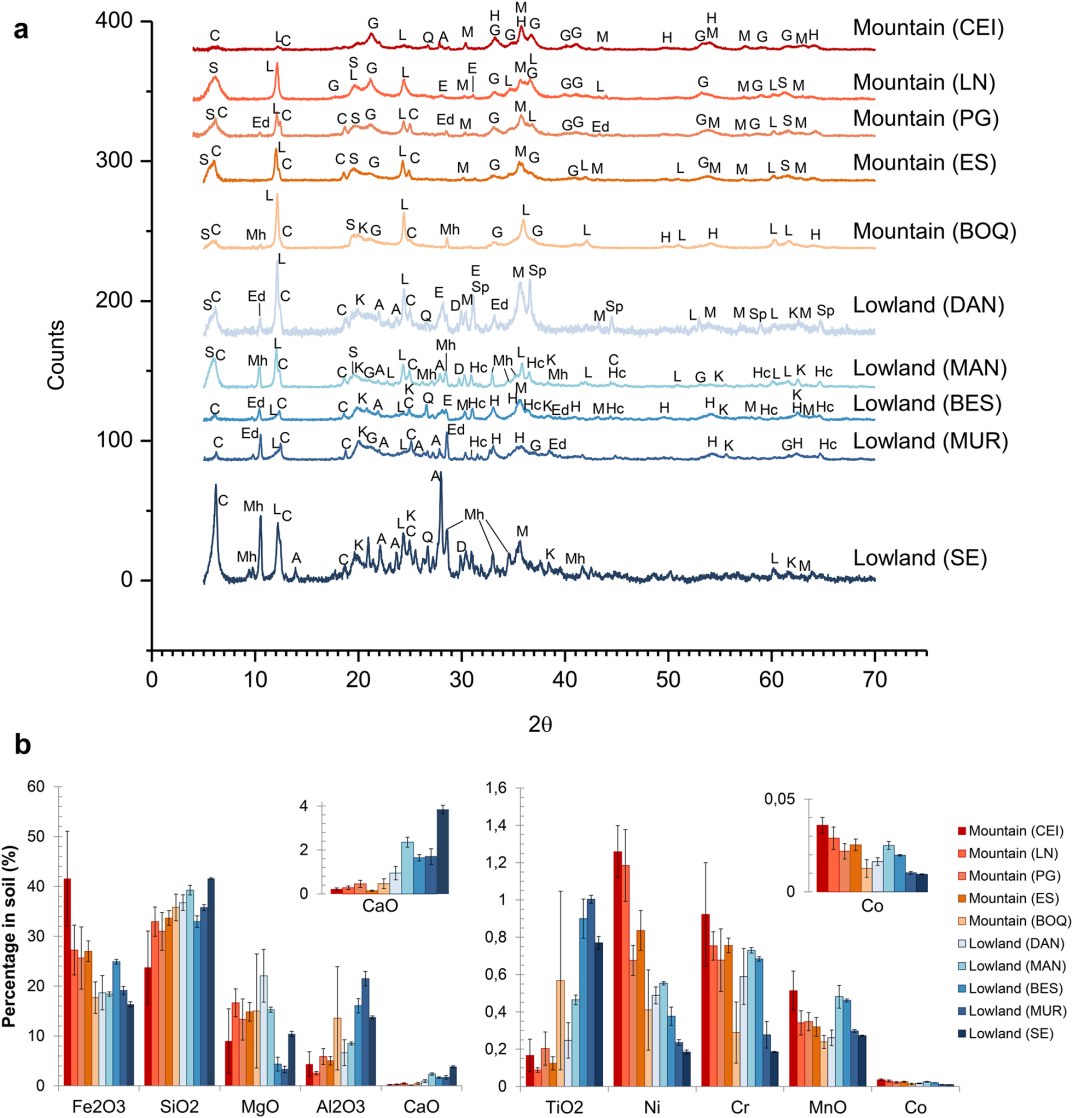
**a** Mineralogy of the serpentine soils from the SEP. Main minerals found with XRD were: lizardite (L), goethite (G), hematite (H), maghemite (M), diopside (D), enstatite (E), hercynite (Hc), ferrite spinel (Sp), clinochlore (C), stevensite (S), magnesio-hornblende (Mh), edenite (Ed), kaolinite (K), quartz (Q) and albite (A). **b** Major elements and main trace metals measured with XRF with their respective standard deviation per location. These 10 elements plus the carbon content (Additional file 1: Table S2) corresponded to 93–98% of the dry mass weight in all the locations sampled. CaO and Co are zoomed in the inset plots. The complete geochemical composition per location is presented in Additional file 1: Table S3

The geochemistry of the soils from the SEP was studied in 10 different locations spread along the eastern, central, northern, and southern areas of the ophiolite and compared with the parent rock geochemistry to analyze the fate of principal metals during soil forming processes. The mineralogy of the soils was determined using powder XRD. All the soils sampled were mainly composed of magnesium silicates, iron oxides and clay silicates confirming the lateritic and serpentine nature of all them, but some differences between locations were observed (Fig. 3a). Lizardite was the only mineral found in all locations although its incidence was considerably higher in the samples from the mountain landscapes, and the lowland samples inside the ophiolite area (DAN and MAN). The mountain locations also shared the presence of iron oxides as maghemite and goethite, and clay minerals such as clinochlore and smectite [(1/2Ca,Na)_0.3_(Mg, Fe, Al)_3_(Si,Al)_4_O_10_(OH)_2_·4H_2_O], the latter closer to a Mg-rich smectite like stevensite [Ca_0.2_Mg_2.9_Si_4_O_10_(OH)_2_·4H_2_O]. However, the BOQ location deviated from the other mountain landscapes as hematite, magnesio-hornblende [Ca_2_(Mg,Fe)_4_AlSi_7_AlO_22_(OH)_2_] and kaolinite [Al_2_Si_2_O_5_(OH)_4_] were present.

In the lowland locations, kaolinite and hornblende-like minerals (close to Mg-rich members as magnesio-hornblende and edenite) and spinel-group minerals were a common characteristic (Fig. 3a). Nevertheless, the lowlands from the inner ophiolite area (DAN and MAN) differed from those at the ophiolite north boundary area (BES, MUR and SE) not only in the lizardite dominancy but also in the presence of smectite minerals. BES and MUR both had hematite as the main iron oxide and a low occurrence of clinochlore, while SE was the most dissimilar sample overall in terms of the mineralogy: dominated by feldspars (albite) and clinochlore, and the only one without spinel minerals. Therefore, the mineralogy of the serpentine soils suggested three main groups of samples, those from the mountains dominated by lizardite and iron oxides, those from inside the ophiolite area dominated by lizardite but with spinel and hornblende minerals, and the others from lowlands to the north boundary of the ophiolite.

The elemental composition was dominated by Fe and Si in all the soils, accounting together for a ~ 60% of the total mass, followed by Mg and Al at between 15 and 25% (Fig. 3b, Additional file 1: Table S3). However, the Fe content was higher in the samples from the mountain landscapes, while Al and Ca had the opposite trend, but with the BOQ soils representing an exception. Mg was considerably lower in the samples from the north limit of the ophiolite (BES, MUR and SE) while the other lowland samples (DAN and MAN) were more similar to the mountain landscapes. In terms of the minor constituents, the concentrations of Ni, Cr and Co were higher in the mountain soils compared to the lowlands, similar to the trend observed with Fe, while Ti had the inverse tendency and Mn was relatively constant across the sites.

When comparing with other serpentinite derived soils worldwide, the Co and Mn contents found here are comparable to serpentine soils from Cuba and New Zealand, and laterites from Australia. However, the soils from the SEP were significantly richer in chromium and nickel when compared with those soils [13, 26, 31, 64]. In two mountain locations (CEI and LN) Ni had values comparable to those of oxide ore Ni laterite deposits [6, 24]. Nevertheless, the content of Fe, Mn, Co, Ni, Cr and Mg were considerably higher than those reported previously for the SEP (Table 2), although one of them considered locations only at the eastern side of the ophiolite and the authors emphasized the small area covered and the lack of biogeochemical information from the rest of the peninsula [31], while other study reported the higher values as abnormalities [65].

**Table 2:** Iron, manganese, cobalt, nickel, chromium and magnesium in geological samples from the SEP

Sample type^a^	Fe_2_O_3_ (%)	MnO (ppm)	Co (ppm)	Ni (ppm)	Cr (ppm)	MgO (%)	Method, Reference
*Serpentine soils (30)*	*14.0–52.1*	*2000–6350*	*70–406*	*1660–13,920*	*1000–12,380*	*2.5–26.4*	*XRF, This study (*Additional file 1: Table S3)
Soils (6)	10.2–16.0 ^b^	1450–2600 ^c^	152–325	3240–7220	1400–3640	3.8–15.6 ^d^	ICP-ES [31]
Soils (8)	< 0.2–6.8 ^b^	11–32 ^e^	18–150 (339) ^f^	24–230 (933, 1047, 2602, 6400)^f^	25–658 (896, 1014, 1670)^f^	2.1–31.2 g	AAS (Mg, Mn) and ICP-ES (Co, Cr, Fe, Ni) [65]
Fluvial sediments (NR)	5.6–6.8 ^b^	560–920 ^c^	34–70	> 100	–	–	AAS [32]
*Serpentinized peridotite rocks (19)*	*7.6–13.5*	*488–2340 *^*c*^	*91–198*	*1759–10,752*	*1870–3480*	*24.6–35.6*	*Bulk XRF, This study*
Peridotite and ultramafic rocks (NR)	–	–	–	1993–2380	1931–2471	34–45	NR [1]
Diabase and basalt rocks (23)	8.9–15.1	130–250	23–37	15–106	30–368	5.0–8.1	XRF (majors) and LA-ICP-MS (traces) [2]

^a^ In brackets the number of samples in the study. ^b^ Fe_2_O_3_, ^c^ MnO and ^d^ MgO were reported as Fe, Mn and Mg % respectively, ^e^ MnO was reported as Mn mg/L after KCl extraction, ^f^ quantities in brackets were reported as abnormal or extreme values, ^g^ MgO was reported as Mg cmol( +)/L after extraction with modified Olsen solution. / NR: Not reported, AAS: atomic absorption spectroscopy, ICP-ES: inductively coupled plasma emission spectroscopy, LA-ICP-MS: laser-ablation ICP mass-spectroscopy

The bulk geochemistry of the serpentine soil samples from all the locations in the SEP, showed analogous patterns to those found in the serpentinzed peridotite parent rocks, with overall positive correlations of Fe, Ni, Co, Mn and Cr (Additional file 1: Table S4, Fig. S7). However, there were significant differences in the extent of positive correlation between the soils and the rocks, especially for Co and Ni. Co had a very strong correlation with Mn in the serpentinite (r = 0.8765, p < 0.0001) but it decreased in the soils (r = 0.7099, p < 0.0001), while its positive correlation with Fe increased from (r = 0.5959, p = 0.0071) to (r = 0.8229, p < 0.0001) respectively. Ni also correlated with Mn although less strongly in the soils (r = 0.3940, p = 0.0312) compared to rocks (r = 0.7175, p = 0.0005). But unlike Co, Ni remained similarly related to Fe in the soils (r = 0.7339, p < 0.0001) as in rocks (r = 0.7790, p < 0.0001). The correlation of Co with Ni was also analogous in the soils (r = 0.8986, p < 0.0001) compared to the rocks (r = 0.8530, p < 0.0001).

The amount of Cr associated with Co, Ni and Mn in soils (r = 0.8951, r = 0.7201, r = 0.7096, respectively, all p < 0.0001) was higher than in the rocks, and in parallel the association with Fe was lower (r = 0.7505, p < 0.0001). On the other hand, the negative correlations of Mg with Fe, Ni and Cr found in the rocks were less significant in the soil. A strong inverse association with soil Al emerged for Co (r = − 0.6862, p < 0.0001), Ni (r = − 0.7775, p < 0.0001) and Cr (r = − 0.6167, p = 0.0003). Finally, a stronger negative correlation between Si and Fe was observed in the soils (r = − 0.9319, p < 0.0001) compared to the rocks (r = − 0.4802, p = 0.0375); a trend also observed for Si with Co, Ni, Cr and Mn (r = − 0.6993, r = − 0.6340, r = − 0.6555, r = − 0.5399, respectively, all with p < 0.002) (Additional file 1: Table S4, Fig. S7).

These results suggest that during serpentine soil formation in the SEP, Co, Ni and Mn are more likely to be concentrated in oxide minerals rather than the hydrous Mg silicate or clay silicate minerals, hence their negative correlation with silicon [24]. Therefore, in the serpentine soils the minerals bearing those elements could be the iron oxides (goethite, maghemite or hematite) and/or the spinel-like minerals (Cr-rich hercynite) according to the overall soil mineralogy (Fig. 3a) and to the mineralogy of the serpentinite clasts where Co was strongly associated with both minerals (Fig. 2e, Additional file 1: Fig. S4-S6). So, during serpentine soil formation, Co, Ni and Mn are concentrated in the Fe-oxides, increasing their correlation with Fe, and in the hercynite too, where Cr is also concentrated. Cr concentration in the spinel is likely to largely substitute for Al, as their strong positive correlation in the rocks is completely inverted in the soils to a negative association; a similar trend occurred with the rocks when comparing serpentinite clasts with less weathered peridotite rocks (see Sect. 3.1) suggesting that Cr-Al substitution in spinels is a characteristic feature of serpentine soil formation during the weathering of serpentinite. However the negative correlation of Fe, Co, Ni, Cr and Mn, with Si could be also strengthened due to the dilution of those metals in a major pool of Si in the soils resulting from the presence of quartz or other alumino-silicates as kaolinite or feldspars that might be the result of the weathering of other lithologies found in the peninsula as gabbros or basalts [4]. This could also explain the strong negative association of Co, Ni and Cr with Al. Therefore, the presence of Co, Ni and Mn in the Mg-rich smectites, clinochlore or amphiboles cannot, be discarded.

### Geochemical-landscape classification of the SEP serpentine soils

**Fig. 4:**
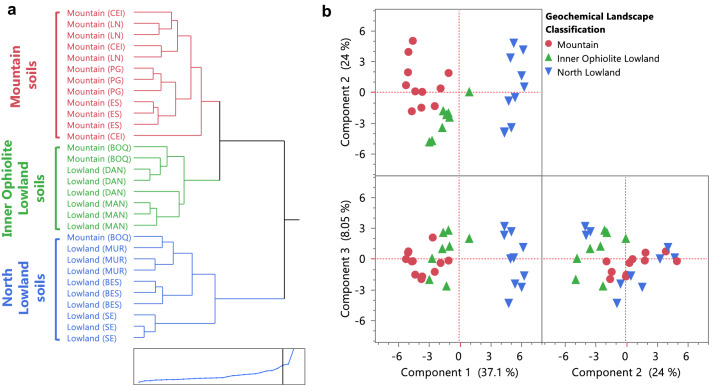
Landscape classification of the serpentine soils from the SEP based on their geochemistry. **a** Three groups of serpentine soils can be considered, top to bottom named as: mountain soils (●), inner ophiolite lowland soils (▲) and north lowland soils (▼). The geographical distribution of each sampling location per cluster can be seen in Fig. 8a, for details within geology, soil and vegetation maps see Additional file 1: Fig. S1. HC considered the total carbon content, the water percentage, pH (Additional file 1: Table S2) and geochemical XRF data (including both majors and traces elements), inset is the distance graph showing the cut point for three clusters. **b** Three main components explained ~ 69% of the variance within the samples according to PCA: PC1 (37.1%), PC2 (24%) and PC3 (8.05%). The score of each variable within each component is in Additional file 1: Fig. S8

Relationships between the location of the samples and their soil geochemistry were studied to establish geographical patterns prior exploring soil metal biogeochemistry. Three distinct groups of serpentine soils could be identified according to their geochemistry: *mountain*, *inner ophiolite lowland* and *north lowland* soils (Fig. 4).

The first group of soils were those from the higher mountain landscapes (CEI, LN and ES) and PG, consistent with their identification as serpentine soils rich in Fe, Ni, Cr and Co in a mineralogy dominated by lizardite and iron oxides. Additionally, within this group of soils, the concentration of Fe, Ni, Mn and Co increased with altitude (Table 3). CEI was the sample collected at the highest altitude and it was the soil with the highest quantities of Fe, Ni, Cr, Mn and Co and the lowest percentage of Si overall, and the smallest content of Mg amongst the mountains, while the soil samples from the LN site, the second highest sampling site, were the closest to the CEI in terms of Ni and Co content (Fig. 3b, Additional file 1: Table S3).

The increasing trend with altitude can be associated with erosive factors and with the low plant cover that was found in those locations. CEI for example, was collected from an area of scarce vegetation and patches of uncovered soils (Table 1, Fig. 1c), and therefore exposed to a higher extent of physical weathering from the local changing conditions of temperature, rain and wind. An analogous case was reported in Italian alpine serpentine soils found above 2000 m altitude, where pedogenesis on serpentinite was very slow or absent when associated with factors such as low plant coverage and steep slopes that favored high erosion rates; resulting in soils with higher amounts of exchangeable Ni compared to most developed soils under coniferous forest [66]. In other non-serpentine soils, like in Galapagos Islands, weathering processes associated with climate were shown to increase with altitude [67] and contributed to larger amounts of secondary phases of Fe, Al and Si at higher elevations [68]. This highlights the necessity to further study the influence of the altitude-associated characteristics (vegetation coverage and climate exposure) in the serpentine soils of SEP despite being the parent rock the main soil-forming agent of these mineral soils [13].

The second group of serpentine soils (Fig. 4) included locations from the *inner* ophiolite area (BOQ, DAN and MAN) (Additional file 1: Fig. S1). This second cluster can be viewed as an intermediate group between the mountain and lowlands originally stated (Table 1), although they were geochemically closer to the mountain soils than to the lowlands sampled in the northern area of the ophiolite (Fig. 4). For example, BOQ was a location in a mountain of relative high altitude (Table 1, Fig. 1) but was highly heterogeneous in its geochemistry yet still with similar composition to the other mountain samples within uncertainties (Fig. 3b), while DAN was a lowland sample inside the ophiolite area which was completely surrounded by high mountains of serpentine landscape like those in the first cluster (Fig. 1, Additional file 1: Fig. S1).

The concentration of metals such as Mn, Cr and Co decreased with altitude (Table 3) in this second cluster of serpentine soils, suggesting that in the ophiolite lowlands enhanced concentrations of metals occur via downslope or downstream mobilization. For example, within this inner ophiolite lowland cluster were also the serpentine soils from the mangrove area of Potrero Grande (Figs. 1c, 4), clustered as a well-preserved group. However, despite being in the same hydrographic basin, MAN and DAN had some geochemical differences, with higher levels of Mg and total carbon upstream (DAN), while Ni, Mn, Co and Cr were more concentrated in the mangrove location (MAN), 10 km downstream from DAN (Table 1, Figs. 1, 3b). A similar trend of concentration of Ni and Cr was reported in mangroves from New Caledonia located downstream a lateritic deposit, with average concentrations of Ni similar to those found in the mangrove from the SEP [69, 70]. Moreover, mangroves acting as metal-sinks or as buffer of metals have been reported, and not only in mangroves downstream lateritic soils such as those from New Caledonia that have high concentrations of Fe and Ni [71]. In other mangroves worldwide within different geological and ecological contexts, metal accumulation occurs, in many cases associated with contamination from anthropogenic activities, such as mangroves in Australia [72, 73], Senegal [74], Singapore [75] or Brazil [76, 77].

**Table 3:** Altitude correlated concentration of elements in the serpentine soils following their Geochemical-Landscape classification (Fig. 4)

Landscape	Element	Alt. Corr	Linear model ^a^	R^2^	F ratio	Prob. > F
Mountain	Fe_2_O_3_	0.6675	Fe_2_O_3_ (%) = 22.25 + 0.032*Alt	0.4456	8.04	0.0177
	MnO	0.6118	MnO (%) = 0.294 + 0.0003*Alt	0.3743	5.98	0.0345
	Ni	0.8606	Ni (%) = 0.65 + 0.0013*Alt	0.7407	28.56	0.0003
	Co	0.8058	Co (ppm) = 204 + 0.30*Alt	0.6493	18.51	0.0016
Inner ophiolite lowland	MnO	− 0.7106	MnO (%) = 0.409–0.0008*Alt	0.5050	6.12	0.0482
	Cr	− 0.8595	Cr (%) = 0.704–0.0012*Alt	0.7387	16.96	0.0062
	Co	− 0.7141	Co (ppm) = 221–0.31*Alt	0.5100	6.24	0.0466

^a^ Graphical model plotted in Additional file 1: Fig. S9./Corr.: correlation, Alt: altitude measured in meters above sea level (masl)

Finally, the last group of soils contained samples from the north boundary of the ophiolite (BES, MUR and SE) (Fig. 4, Additional file 1: Fig. S1). In general, these were soils from lowlands poor in Mg but rich in Al, Ca and Ti (Fig. 3b). Their geochemical composition together with the mineralogy reflected not only a serpentine nature but more complex weathering and erosive processes involved in their formation, probably due to their geographical position between different geological units (Additional file 1: Fig. S1), but collectively still influenced by the laterite geochemistry of the ophiolite mountains (reflected in the lizardite common mineral). However, BES and MUR were slightly different to SE, relying mainly on a higher amount of Si, Ca and Na (Fig. 3b), and reinforced by the different mineralogy dominated by albite in this location (Fig. 3a), thus coinciding with the soil type separation previously reported for the SE site (Table 1, Additional file 1: Fig. S1).

In summary, although all the soils sampled were clearly lateritic, there were measurable variations in their geochemical composition, resulting in three types of serpentine soils according to their landscape and geographical location. Two groups of soils, having more serpentine-like characteristics, were present within the ophiolite area. In the ﻿*mountain*﻿ soils the concentrations of Fe and trace elements including Co, Ni and Mn were higher, and the concentration of Fe, Mn, Ni and Co increased with the altitude. In the piedmonts and the valleys between those serpentine-soil-mountains located inside the ophiolite area, the *inner ophiolite lowland* soils retained characteristics from the surrounding highlands, and the concentration of the trace metals studied such as Cr, Co and Mn increased downstream, accumulating in the mangrove area. The *north lowland* soils at the northern boundaries of the ophiolite had the most dissimilar geochemistry nevertheless could be considered as serpentine soils because were still influenced by the ophiolite but to a lesser extent than the other two types of serpentine soils. Although the geographical-geochemical patterns found here could be later refined considering additional samples with other ecological traits (climate exposure, vegetation, depth variations, soil-forming properties, etc.) or from inaccessible and thus unexplored areas of the National Park, this classification will serve as the foundation to further explore local biogeochemical inputs to the serpentine ecosystem.

### Microbial characterization of the serpentine soils

The microbial composition of the serpentine soils associated with the SEO was studied to explore groups of microorganisms that could be implicated in potential biogeochemical cycling of metals occurring in those soils according to local geochemical patterns previously described here. The microbial characterization was done by sequencing the V4 variable region of the 16S rRNA gene for the prokaryotic communities and the ITS2 region of nuclear ribosomal DNA for fungi. To our knowledge, this is the first report of both the prokaryotic and fungal communities of the soils along the SEP. Only two previous studies have reported indigenous microorganisms of this area, but both were restricted to methane-related prokaryotes in three hyperalkaline springs [34, 35].

#### Prokaryotic communities in the soils

**Fig. 5:**
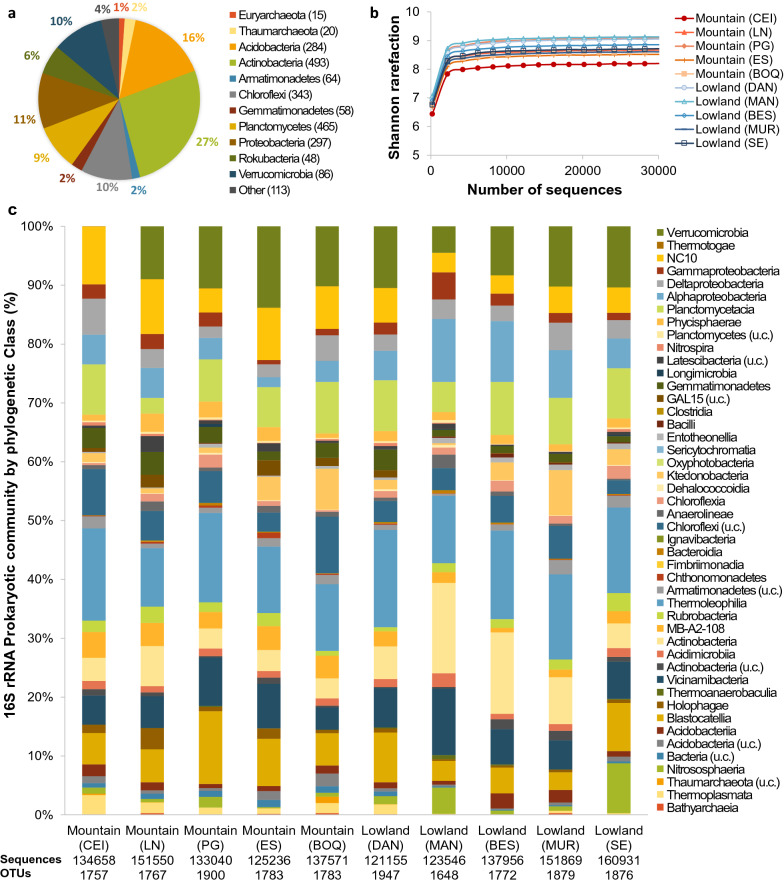
Prokaryotic communities in the serpentine soils from the SEP obtained by the sequencing of the V4 region of 16S rRNA and considering the two main landscapes present (Table 1). **a** Relative abundance per phylum of all the sequences obtained in the entire sampling area associated with the SEO. In brackets the number of prokaryotic phylotypes (OTUs). “Other” included Crenarchaeota (7 OTU, 1795 sequences), Bacteroidetes (20 OTU, 3205 sequences), BRC1 (1 OTU, 94 sequences), Cyanobacteria (2 OTU, 261 sequences), Entotheonelleota (8 OTU, 7601 sequences), Firmicutes (2 OTU, 718 sequences), GAL15 (10 OTU, 10,863 sequences), Latescibacteria (27 OTU, 11,142 sequences), Nitrospirae (6 OTU, 3598 sequences), Thermotogae (1 OTU, 145 sequences), and unclassified Bacteria (22 OTU, 8782 sequences). **b** Shannon rarefaction curve. **c** Prokaryotic communities per phylogenetic class in each location sampled. Total OTUs obtained: 2286, total sequences read: 1,377,510; u.c.: unclassified class

Regardless of the location or the landscape considered, the prokaryotic communities in all 10 samples studied appeared broadly similar in their composition at the phylum level (Fig. 5). The prokaryotic diversity was very similar among the sites as showed in rarefaction curves, although the communities were slightly less diverse in the mountain CEI sample (Fig. 5b, Additional file 1: Fig. S10), and in terms of the phylotype richness, there was no clear distinction between the two main landscapes originally proposed in Table 1 (F = 0.3173, p = 0.5887). The prokaryotic communities in general were clearly dominated by Bacteria (Fig. 5a), with only 3% of the total abundance corresponding to Archaea.

The Archaeal domain was represented by three phyla and 42 phylotypes, 20 were from the Thaumarchaeota (2% of the total relative abundance), 15 phylotypes were affiliated with the Euryarcheota (1% relative abundance) and 7 with the Crenarchaeota (0.1%). Both major groups found are commonly present in soils worldwide, but Euryarcheota has been reported principally in water-saturated soils [78–80]. When considering every location sampled (Fig. 5c), Thaumarcheota were ubiquitous along the peninsula but higher in in the mangrove location (4.5% relative abundance) and in the most geochemically dissimilar lowland sample (SE) (8.5% relative abundance), probably involved in nitrogen cycling in those sites, as this group comprises ammonia-oxidizing microorganisms [79, 80]. Euryarcheota were represented by microorganisms affiliated with the order Methanomassiliicoccales (Thermoplasmata class) and were more abundant in all the mountain locations (1–3% relative abundance) and the lowland soils from inside the ophiolite area (DAN) (2% relative abundance). The representatives of this order have been reported as methanogens in several environmental contexts such as rice paddy fields, wetlands, digestive tracts of animals or low sulphate wells [81, 82].

Actinobacteria represented the major bacterial phylum within the serpentine soils in the SEP both in phylotype richness (493) and total abundance (27%), followed by representatives of the Acidobacteria (284 OTUs and 16% total abundance) and Proteobacteria (297 phylotypes, 11% of total abundance) (Fig. 5c). Those three phyla are also the dominant clades in soils across the world although Proteobacteria tend to be the dominant group [83]. In every location (Fig. 5c), Actinobacteria was also the major phylum detected (including Acidimicrobiia, Actinobacteria, Rubrobacteria and Thermoleophilia classes). However, is notable that this group was less abundant in the mountain landscapes (21–25% relative abundance) compared to the lowlands (26–34%). Nonetheless, these levels were considerably higher than those reported from other ultramafic soils in New Caledonia, where Actinobacteria only accounted for 8–10% in largely Proteobacteria-dominated soils [84]. This group is also one of the largest clades recognized in manganese-rich soils, and includes numerous manganese-oxidizing bacteria [85, 86], several of which were found in all the locations of the peninsula such as *Mycobacterium*, *Pseudarthrobacter*, *Actinophytocola*, *Pseudonocardia*, *Saccharopolyspora* and *Streptomyces*. Generally, these Mn cycling-related taxa were more abundant in the lowland landscape soils (3–9% relative abundance) rather than in the mountain samples (2–4%). The Mn-enriched mangrove (MAN) sample (Fig. 3b) contained the largest relative abundance (9%) of these Mn-oxidizing taxa (Additional file 1: Fig. S11), with *Streptomyces* being the largest clade present (2.6%), a genre that has been reported as highly abundant in manganese-contaminated mine tailings [86]. Many of these Actinobacteria have been also associated with non-tropical ultramafic soils rich in nickel and chromium in Spain [87].

The other group of interest potentially associated with manganese oxidation was the second most common clade overall, Proteobacteria, including species of *Pedomicrobium* (Alphaproteobacteria), *Pseudomonas* and several of the Burkholderiaceae (Gammaprotobacteria) [20, 85, 86, 88]. In general, these groups represented 0.7–3.3% of the relative abundance per location, and similarly to the taxa mentioned before, the higher abundance was found in the mangrove (Additional file 1: Fig. S11). Additionally, Firmicutes is another clade that has been associated with manganese cycling [85, 86], and although it was a minor group in the serpentine soils of Santa Elena, *Bacillus* species affiliated with this phylum were found in all the locations; better represented in the lowlands (0.2–0.7% of relative abundance, except DAN with < 0.05%) compared to the mountain locations (< 0.1% of relative abundance).

Sequences closely related to known iron-reducing bacteria were identified, including Deltaproteobacteria such as *Geobacter* or *Anaeromyxobacter* [89–91]. However, *Anaeromyxobacter* was more abundant, being in larger proportions in the highest mountains (Additional file 1: Fig. S11). In fact, the highest proportion of *Anaeromyxobacter* was found in the CEI mountain location (4% relative abundance); the site with the greatest quantity of iron (Fig. 3b). Well-known iron-oxidizing bacteria were also present at the different locations (Additional file 1: Fig. S11), including those affiliated with the manganese-oxidizing Gammaproteobacteria mentioned before (that can also oxidise Fe(II)) [92], and phylotypes from the Nitrospira class [93].

Finally, other bacteria found whose functionality could be inferred included those associated with the nitrogen cycling, such as those from the order Nitrospirales (within the Nitrospira class) (Fig. 5c) [93]. Nitrogen cycling bacteria were relatively ubiquitous across all the locations, complimenting potential ammonia oxidizing archaeobacteria described previously (Additional file 1: Fig. S11). Sulphur-cycling Deltaproteobacteria from the orders Desulfarculales and Desulfuromonadales were found at all the locations (0.4 1.3% relative abundance) (Additional file 1: Fig. S11) [91, 94].

#### Fungal communities in the soils

**Fig. 6:**
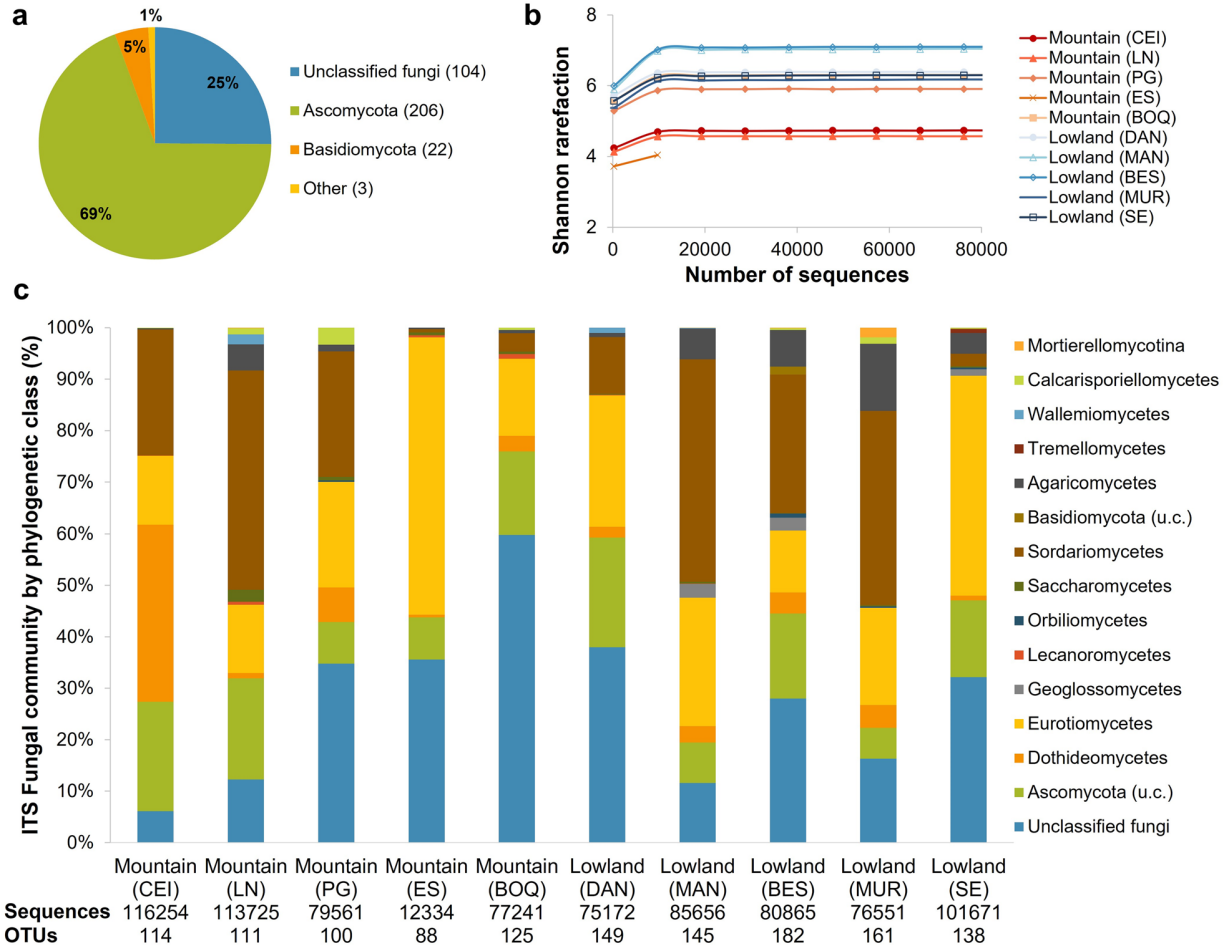
Fungal communities per phylogenetic class in the serpentine soils of the SEP obtained by ITS2 sequencing and considering the two main landscapes present (Table 1). **a** Relative abundance of all the sequences obtained per phylum in the entire sampling area associated with the SEO. In brackets the number of fungal phylotypes (OTUs). "Other" included Calcarisporiellomycota (1 OTU, 5923 sequences) and Mortierellomycota (2 OTU, 1671 sequences). **b** Shannon rarefaction curve. **c** Fungal communities per phylogenetic class in each location sampled. Total OTUs obtained: 338, total sequences read: 818,985; u.c.: unclassified class

The fungal communities in the serpentine soils were also studied, by sequencing the ITS2 region of nuclear ribosomal DNA (Fig. 6). In terms of the phylotype richness, soils from the lowland landscapes were richer and had more OTUs (155 ± 7) than those from the mountains (108 ± 7) (F = 22.61, p = 0.0014) (Fig. 6b, Additional file 1: Fig. S12), with the mountains CEI and LN the less diverse locations while lowlands BES and MAN were more diverse. In general, none of the locations were as rich or diverse as their prokaryotic communities (Fig. 5b, Additional file 1: Fig. S10). Over all the soils sampled across the SEP, the fungal communities were Ascomycota-dominated with 206 OTUs (61% of the total OTUs found) representing 69% of the total sequences against 22 OTUs that were basidiomycetes (5% total sequences), and with a high percentage of unclassified fungi (104 OTUs, 25% total sequences) (Fig. 6a). Most of the locations had a similar distribution of phyla when compared to the complete peninsula, but three sites had much higher percentages of ascomycetes: the mountains CEI and LN (93% and 79% of relative abundance, respectively), and the mangrove location MAN (82%) (Fig. 6c). Those were also the locations with fewer unclassified fungi, although they had the larger quantities of unclassified Ascomycota (Fig. 6c). The mountain BOQ had the largest proportion of unclassified fungi, and the fewest Ascomycota (31% of relative abundance). Large numbers of unclassified fungi could be explained both by the high unexplored diversity normally found in the tropics, where the ACG in particular is considered a hotspot of biodiversity, and the expected endemism associated with serpentine soils due to their high relative levels of trace metals and general abiotic stress; similar to the endemism broadly reported in plants from serpentine ecosystems [16, 84].

Amongst the ascomycetes, Dothideomycetes was the largest phylogenetic class found in the mountain CEI location (34% of relative abundance), much higher than any other site (Fig. 6c). This clade was also a major fungal constituent in laterites rich in Co and Ni from Brazil and Phillipines [25]. In Santa Elena, this group was mostly represented by Mycosphaerellaceae, where many species are described as plant pathogens, although their members can also cover other niches and lifestyles [95]. Interestingly, the serpentine soils from the mountain CEI location were covered with Poaceae plants (grasses), a family also associated with certain species of Mycosphaerellaceae [96]. However, these grasses were also present in the other mountain locations, but these fungi were absent regardless of their vegetation or altitude. As noted previously, the other main difference between the CEI and the other mountain soil samples were the highest levels of iron and trace metals overall, suggesting a more complex association between these fungi, the local plants and the soil geochemistry that should be further addressed.

Eurotiomycetes were present in all the soils sampled (Fig. 6c), including species of *Aspergillus* and *Penicillium* as the most common constituents. *Aspergillus* and *Penicillium* are fungal genera commonly isolated from heavy metal polluted areas [97] and were also found in other Co and Ni-rich laterites [25]. This may suggest a relationship with the geochemical nature of the serpentine soils of the SEP, where the mountain soils have large proportions of certain trace elements, while the mangrove was geochemically distinct to the other lowlands due to its metal-accumulation characteristics as said before. *Aspergillus* for example, had slightly higher proportions in the mangrove sample (MAN) (12% of relative abundance) compared to the mountains (PG, ES, CEI and LN) (4–10% of relative abundance). However, in contrast to this suggestion, *Penicillium* species were more common in the lowlands compared to the mountains, with 2–11% of relative abundance, suggesting the exploitation of other ecological niches (except mountain ES that could be an underrepresented sample).

The Sordariomycetes were the third important class of ascomycetes found, although their subsequent classification varied depending on the locations (Fig. 6c). The Coniochaetales were the dominant Sordariomycetes in the mountains CEI and LN and restricted to those two sites (21% and 37% of relative abundance respectively); a group that has been reported from different soils [98]. The Hypocreales were represented by *Fusarium* (4–11% of relative abundance) and *Purpureocillium* (16–21%) in the lowland soils except in the location upstream in the ophiolite area (DAN) and the one to the northwest (SE) (Additional file 1: Fig. S1). *Fusarium* is also commonly isolated from heavy metal polluted areas [97] and was identified in Ni and Co-rich laterite sediments, same as *Purpureocillium* [25]. Moreover, *Purpureocillium* has been reported as endophyte in mangrove plants, it was shown to induce the complexation of Cu, Mn and Fe in the soil [99]. This highlights the necessity to better understand the role of this fungal genus especially towards the mangrove area of Potrero Grande here studied. Location PG had an analogous content of *Purpureocillium* (11%), but this was the mountain site with the lowest altitude and the one with the most different vegetation leading towards the type found in the lowland locations (Table 1, Fig. 1). All these results suggest an interesting relationship of the vegetation type and the presence *Purpureocillium* within the serpentine system. The influence of vegetation cover in the microbial diversity of prokaryotes and fungi has been reported in ultramafic soils in New Caledonia, an issue of high interest to further study in the SEP [84, 100, 101].

Finally, the other important fungal phylum in the soils of the SEP was the Basidiomycota, which was better represented in the lowland locations (5–13% of the total abundance) (Fig. 6c). The lowland location inside the ophiolite area (DAN) had a lower abundance of basidiomycetes (2%) while the mountain location LN had a similar proportion to the other lowlands (7%), and all of them were dominated by Agaricomycetes. An interesting case was the presence of the macrofungi *Lycoperdon* in mountain LN and lowland MAN (5% of relative abundance in each location), a fungal genus reported to accumulate metals such as gold, mercury, lead, zinc, copper, iron, manganese chromium, nickel and cobalt [97, 102, 103].

### Integrating the geochemistry and microbiology of the serpentine soils in a landscape context, implications to metal biogeochemistry

The association of geochemical and microbial data obtained for the serpentine soils of the SEP provide evidence for potential impacts on local metal biogeochemical cycles. For example, two trends warrant comment in a context of metal biogeochemistry: (1) correlation between Fe and Co increased in the soils and (2) Ni and Co in the serpentine soils had a weaker correlation with Mn when compared with the serpentinite clasts, suggesting that Mn could have been leached to some extent from the Fe-oxide minerals leading to the concentration of Ni and Co. Any of these processes could be associated with geomicrobiological processes because microbes are known to weather Mn and Fe minerals, with Mn-Co redox cycling reported in laterites too [20, 21, 25].

The prokaryotic communities in the serpentine soils of the SEP exhibited characteristics that related to the geochemistry of their site of origin despite an apparent homogeneity across the sampled area of the ophiolite. The mountain and lowland soils shared many phylogenetic groups in similar proportions, however those related to iron redox cycling were more abundant in the mountain landscapes compared to the lowlands. The mountain CEI location had the highest relative abundance of iron reducers and the lowest diversity overall, while the lowland sample from the inner ophiolite area (DAN) surrounded by mountains had microbial communities analogous to the mountain landscapes. The lowlands were richer in potential manganese-oxidizing bacteria, especially those from Actinobacteria, where the mangrove location had the greatest abundance. These results were analogous to the geochemical relationships between the locations sampled, as higher concentrations of Fe and minerals of Fe(III) were found in the mountain soils, whilst the mangrove was one of the locations with the highest concentrations of Mn, reflecting the interdependence of the prokaryotic communities and the geochemistry of the serpentine soils.

Additionally, the composition of the fungal communities per location also complimented the results previously described in the geochemistry of the serpentine soils of the SEP. Amongst the mountains, CEI and LN were the two locations with the highest amounts of iron and trace metals, and they also had a different fungal structure evident from the presence of Coniochaetales, restricted to those two locations, and the largest proportion of Dothideomycetes in CEI. The sample from the mangrove (MAN) had a distinct fungal composition compared to the location upstream (DAN), reflecting the differences in their geochemical characterization. The mangrove behaved as a metal-sink downstream and metal-related clades such as *Aspergillus*, *Fusarium* and *Purpureocillium* species were abundant there. More generally, several fungal groups that have been previously associated with metal cycling were also identified in these metal-rich serpentine soils, highlighting the potential importance of fungal communities within the serpentine system as modulators of metal content and the close relationship with vegetation (an area for future research interest).

**Fig. 7:**
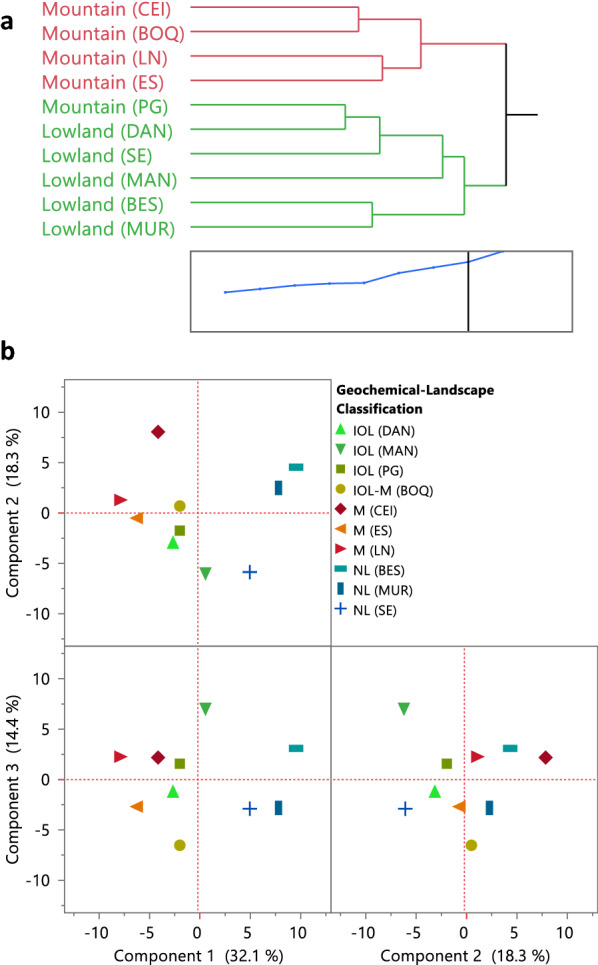
Microbial-geochemistry associations in the serpentine soils from the SEP. **a** Landscape classification of the serpentine soils based on their microbial composition, considering the abundance of prokaryotes and fungi per phylogenetic class, and soil geochemistry. Inset is the distance graph showing the cut point for two clusters. **b** Microbial-geochemistry associations based on the Geochemical-Landscape classification of Fig. 4 and considering prokaryotic and fungal abundance for every location sampled. Three main components explained ~ 65% of the variance within the samples according to PCA: PC1 (32.1%), PC2 (18.3%) and PC3 (14.4%). The score of each variable within each component is in Additional file 1: Fig. S13

When considered together, these data confirmed the complex processes underpinning the mobilization of trace elements during serpentine soil formation in Santa Elena, probably involving both geochemical and biological factors in different stages of their respective metal biogeochemical cycles. Therefore, prokaryotic and fungal data were analyzed using hierarchical clustering analysis to study the relationships between the microbial communities and the geochemistry of these serpentine soils (Fig. 7).

Two major groups were evident (Fig. 7), the first cluster included all the locations with mountain landscape except the one at the lowest altitude (PG) (Table 1), which was clustered with all the lowland samples. This separation could be related to the vegetation because it corresponds, except for PG location, to the landscape differentiation observed in the SEP (Table 1, Fig. 1) that was proposed using vegetation type as one of the main characteristics of classification. PG was the only mountain location that was not grass-dominated and had taller trees as can be seen in Fig. 1, a vegetation more similar to the DAN lowland at the inner ophiolite area; coinciding with the closest location to PG according to Fig. 7a. Those results suggest that vegetation (linked to altitude) might be an important driving force in shaping the microbial communities of the serpentine soils of SEP. Vegetation strongly influences microbial communities, including both prokaryotes and fungi, in tropical serpentine soils [84, 100, 101], non-tropical serpentine soils [66, 87, 104] and non-serpentine soils in general [83]. However, the differentiation of microbial communities can be associated with abiotic factors too. For example, altitudinal differences in soil microbial communities were found in subalpine forests where riparian zones and upland zones differed according to the soil water content [105]; and more generally, pH of soils and soil geochemistry can drive microbial communities both in serpentine and non-serpentine soils [83, 84, 100, 106].

**Fig. 8:**
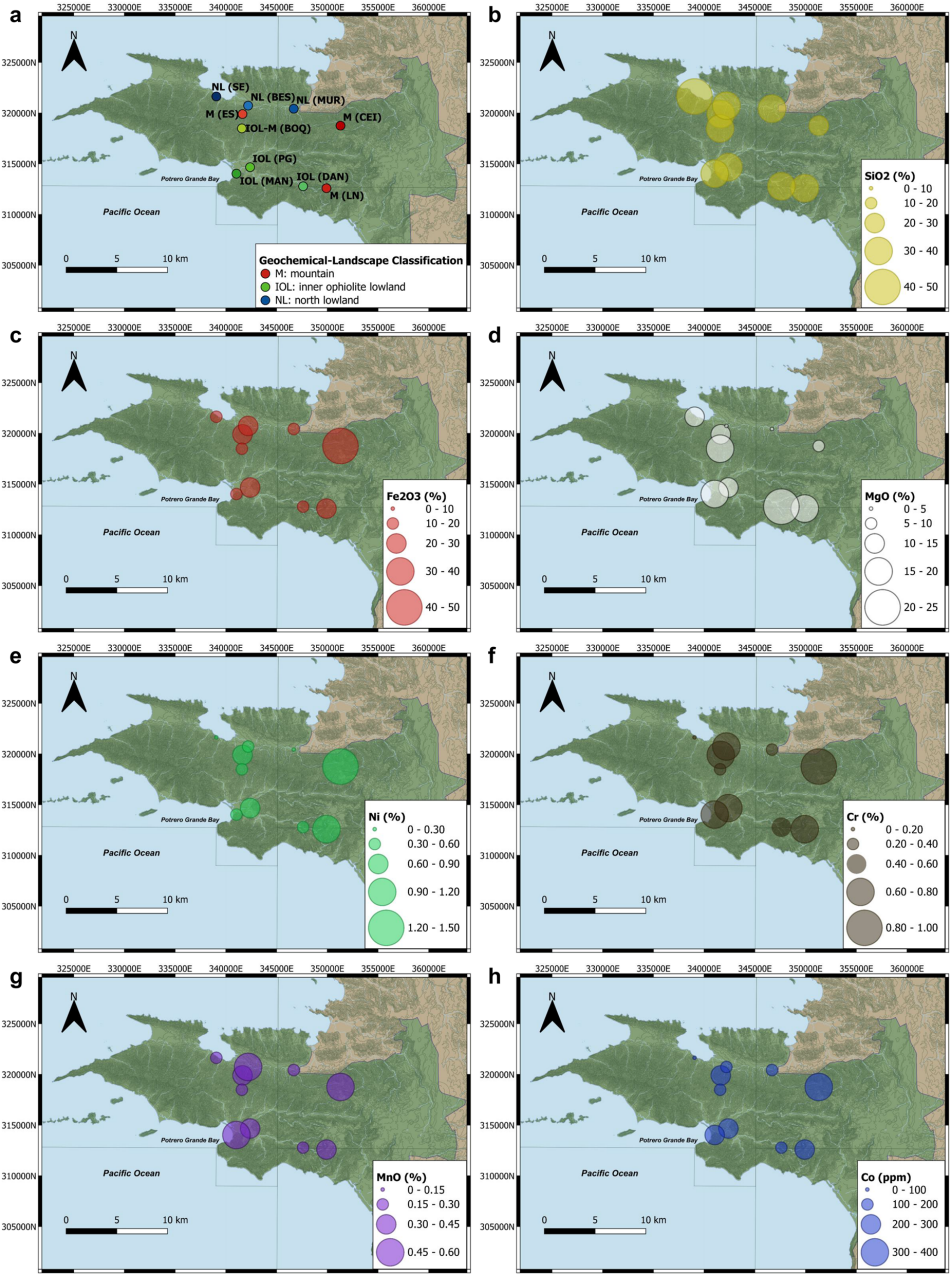
**a** Biogeographical distribution of the three serpentine soil groups found in the SEP based on biogeochemistry and geomicrobiology (see Figs. 4, 7), and the distribution of major elements: Si (**b**), Fe (**c**) and Mg (**d**), and key trace metals of geomicrobiological interest: Ni (**e**), Cr (**f**), Mn (**g**) and Co (**h**)

The relatively large compositional similarities between locations sampled both in the prokaryotic and fungal communities of the serpentine soils of SEP (Figs. 5, 6) produced clusters for the microbial-landscape distribution not as distantly separated (Fig. 7a) as in the geochemistry-based classification (Fig. 4); however, some biogeochemical relationships warrant comment. The mountain cluster represents a well-preserved group of soils characterized by a relatively high Fe and trace metals content such as Ni, Co, or Cr, and therefore microorganisms associated with the iron redox cycling (Figs. 3b, 7, 8). The geochemistry-based locations from the inner ophiolite lowland soils (BOQ, DAN and MAN) and the lowest mountain soil (PG) (Fig. 4) had mixed behaviors when considering the microbial communities. BOQ behaved similarly to the other mountain locations to which was also geochemically close; DAN, and PG, on the other hand had a closer microbial composition to that of the other lowland samples but their geochemistry was closer to that of the mountains (Figs. 7, 8). Moreover, as said before the closeness between PG and DAN locations could be due to the similarities in altitude and vegetation between both sites, highlighting the close association between altitude, vegetation, geochemistry, and microbial communities in these serpentine soils. Therefore, in terms of the soil geomicrobiology, PG and DAN should be considered as a separate group, with geochemical and microbial characteristics between the mountain and the lowland landscapes originally proposed, and thus, biogeographically, here are proposed as the “inner ophiolite lowland” serpentine soils of the SEP (Fig. 8a).

Furthermore, the mangrove location (MAN) emerged as an interesting place, geochemically closer to the other sites of the inner ophiolite lowland group (Fig. 4) but geomicrobiologically closer to the other lowlands (Figs. 7, 8). Here, the Mn concentration stands as a trait of potential ecological and biogeochemical importance. Also, within the same riparian basin the composition of the microbial community upstream (DAN) was relatively dissimilar to the mangrove location (MAN) downstream (Figs. 7, 8). The geochemical composition between DAN and MAN differed to some extent in certain characteristics despite being in the same geochemical cluster (Fig. 4) but the vegetation was an evident contrasting trait amongst both sites (Table 1, Fig. 1). Thus, the dissimilarity found in this basin of SEP suggested that vegetation could have a stronger influence driving soil microbial communities than the geochemical characteristics of the serpentine soils, a trend also observed in other tropical serpentine soils from New Caledonia [84, 100]. Finally, the lowland towards the northern boundaries of the ophiolite area, BES and MUR locations were more similar to each other than to SE both geochemically and microbiologically (Figs. 7, 8).

In summary, these results evidence how microbial communities in the serpentine soils of the SEP are linked and subjected to the variations of an intricate ecological network of both biotic factors like vegetation type and coverage, and abiotic factors such as the geochemistry of the soils or the altitude, even within a small geographical area. These relationships must be reflected within the biogeochemical cycling of metals found in the serpentine soils, but further research is required to better understand which mechanisms are involved, and their overall impacts (including other locations within the SEP and variables such as climate exposure, depth variations, soil-forming properties, etc.). Thus, despite the unique ecological characteristics of this area, the SEP demonstrated a great potential as a model site to study serpentine ecosystems in tropical conditions and complex metal biogeochemical cycles within various ecological and landscape contexts.

## Conclusions

The geochemistry of the serpentine soils associated with the area of the SEO was characterized considering a landscape-biogeographical approach. The soils were Ni-rich laterites, but with geochemical variations depending on their geographical position within the ophiolite area, reflecting differences in the degree of serpentinization in soil-hosted rock clasts associated with different in situ weathering processes, and thus resulting in three different serpentine soil types: *mountain* soils, *inner ophiolite lowland* soils and *north lowland* soils. The mountain soils were dominated by lizardite and iron oxide minerals, and the influence of altitude-associated characteristics, as the minor vegetation coverage and thus more direct climate exposure, favored the concentration of Fe, Mn, Co and Ni in these soils. The serpentine soils in the inner ophiolite lowland areas were geochemically closer to the serpentine soils present in the surrounding mountains, and within the same riparian basin the concentration of trace metals increased downstream with higher concentrations towards the mangrove, acting as a metal-sink area. The north lowland soils, despite sharing the soil taxonomic classification type with other lowlands, were the less serpentine-like group of soils although were still influenced by the ophiolite geochemistry to a lesser extent. Therefore, when studying the serpentine soils of the SEP with a biogeochemical focus, the geochemical composition of the soils -and not only the soil taxonomic classification- and the geographical location within the ophiolite area should be considered, especially in inner ophiolite lowland areas or in the top of the mountains.

The native microbial communities of these serpentine soils were also studied and contrasted against soil geochemistry, revealing potential geomicrobiological associations that could impact the biogeochemistry of metals in the site. In the mountain locations, richer in Fe and associated trace metals, the abundance of prokaryotes related to Fe-redox cycling was higher. In the lowlands Mn-oxidizing bacteria were more abundant, and two groups of lowland soils were identified supporting the geochemical-landscape classification proposed for the serpentine soils of the SEP. However, the mangrove area of Potrero Grande, was microbially distinct when compared to its upstream location, also registering the major abundance of Mn-oxidizing bacteria overall and a distinct fungal community. Although the microbial communities of the serpentine soils could be associated with the soil geochemistry, vegetation coverage and altitude could have an important influence driving the microbial communities too, and this must be further studied. Also, large amounts of unclassified organisms were found, presenting the SEP as a potential hotspot of serpentine specialism and endemism.

This research proved the SEP as a place to study the natural relationships between the geochemistry and the microbial communities of serpentine soils and will serve as a basis for future work to better understand the biogeochemical cycles of metals occurring there. Moreover, the landscape-biogeographical focus of this study evidenced the complex associations between microbial communities, vegetation, altitude and the geochemistry of soils under active laterite formation processes. Therefore, in a wider approach the SEP emerges as a potential model site to understand the natural interactions between biotic and abiotic factors occurring in tropical serpentine ecosystems, and more generally, to study the natural biogeochemical cycles of metals such as Fe, Ni, Co, Mn or Cr, under different ecological and landscape contexts.

## Supplementary Information

**Additional file 1: Fig. S1.** Geographical distribution of the serpentine soils sampled in 10 locations of the SEP within the maps of the geology (a), soil taxonomy (b) and vegetation (c). **Fig. S2.** Bulk elemental correlations in serpentinized rocks from the SEP for Fe_2_O_3_, Mn, Co, Ni, Cr, MgO, Al_2_O_3_, and SiO_2_, all of them analyzed by XRF. **Table S1.** Linear correlations and probabilities associated for Fe_2_O_3_, Mn, Co, Ni, Cr, MgO, Al_2_O_3_, and SiO_2_ found in serpentinized rocks from the SEP, all of them analyzed by XRF. **Fig. S3.** Bulk mineralogy of rock clasts from the soils (10 cm depth) of the SEP. **Fig. S4.** Elemental distribution determined on a polished thin section of a serpentinite clast from 10 cm depth (Lowland site MAN), defined by EPMA. **Fig. S5.** Elemental distribution determined on a polished thin section of a serpentinized peridotite from an outcrop close to Mountain CEI location, defined by EPMA. **Fig. S6.** Elemental distribution determined on a polished thin section of a serpentinized peridotite from an outcrop close to Lowland BES location, defined by EPMA. **Table S2.** Water content, total carbon content and pH of the soils sampled. **Table S3.** Elemental geochemical composition of the lateritic/serpentine soils from 10 locations of the SEP, analyzed by XRF majors (%) and traces (ppm). **Fig. S7.** Bulk elemental correlations in the serpentine soils from the SEP for Fe_2_O_3_, Mn, Co, Ni, Cr, MgO, Al_2_O_3_, and SiO_2_, all of them analyzed by XRF. **Table S3.** Linear correlations and probabilities associated for the Fe_2_O_3_, Mn, Co, Ni, Cr, MgO, Al_2_O_3_, and SiO_2_, found in serpentine soils from the SEP, all of them analyzed by XRF. **Fig. S8.** Principal components analysis (PCA) for the geochemistry of the serpentine soils and the score of each variable within each component. **Fig. S9.** Altitude correlation of the concentration of Fe (a), Mn (b), Ni (c) and Co (d) in the mountain serpentine soils and of Mn (e), Cr (f) and Co (g) in the inner ophiolite lowland serpentine soils. **Fig. S10.** Rarefaction curves for the prokaryotic communities in the serpentine soils overall the locations sampled in the SEP per observed species (a) and Fisher Alpha (b) after sequencing the V4 region of 16S rRNA. **Fig. S11.** Abundance of sequences per prokaryotic metabolic function assigned to phylogenetic clades after sequencing the V4 region of 16S rRNA in the serpentine soils overall the locations from the SEP. **Fig. S12.** Rarefaction curves for the fungal communities in the serpentine soils overall the locations sampled in the SEP per observed species (top) and Fisher Alpha (bottom) after sequencing the ITS2 region of nuclear ribosomal DNA**. Fig. S13. **Principal components analysis (PCA) for microbial-geochemical associations in the serpentine soils from the SEP and the score of each variable within each component.

## Data Availability

The datasets supporting the conclusions of this article are available in https://doi.org/10.17632/w6cdt3sn99.4 with CC BY 4.0 license, in NCBI SRA (Sequence Read Archive; http://www.ncbi.nlm.nih.gov/sra/, under the project accession number: PRJNA606410) and within the article and its additional file.

## Abbreviations

SEP: Santa Elena Peninsula
ACG: Área de Conservación Guanacaste (Guanacaste Conservation Area in Spanish)
SEO: Santa Elena Ophiolite
UNESCO: The United Nations Educational, Scientific and Cultural Organization
XRD: X-ray diffraction spectroscopy
XRF: X-ray fluorescence spectroscopy
LOI: Loss on ignition
EPMA: Electron probe micro analysis
GIS: Geographic information system
HC: Hierarchical cluster analysis
PCA: Principal component analysis
DNA: Deoxyribonucleic acid
rRNA: Ribosomal ribonucleic acid
ITS: Internal transcribed spacer
PCR: Polymerase chain reaction
OTU: Operational taxonomic units
AAS: Atomic absorption spectroscopy
ICP-ES: Inductively coupled plasma emission spectroscopy
LA-ICP-MS: Laser-ablation inductively coupled plasma mass-spectroscopy
