# Supplementary material for: Geochemistry and microbiology of tropical serpentine soils in the Santa Elena Ophiolite, a landscape-biogeographical approach
Source: Geochem Trans. 2022 Sep 27;23:2. doi: 10.1186/s12932-022-00079-5 (PMC9516835; doi:10.1186/s12932-022-00079-5)
Supplement: Supplementary file 1 — Additional file 1: Fig. S1. Geographical distribution of the serpentine soils sampled in 10 locations of the SEP within the maps of the geology (a), soil taxonomy (b) and vegetation (c). Fig. S2. Bulk elemental correlations in serpentinized rocks from the SEP for Fe2O3, Mn, Co, Ni, Cr, MgO, Al2O3, and SiO2, all of them analyzed by XRF. Table S1. Linear correlations and probabilities associated for Fe2O3, Mn, Co, Ni, Cr, MgO, Al2O3, and SiO2 found in serpentinized rocks from the SEP, all of them analyzed by XRF. Fig. S3. Bulk mineralogy of rock clasts from the soils (10 cm depth) of the SEP. Fig. S4. Elemental distribution determined on a polished thin section of a serpentinite clast from 10 cm depth (Lowland site MAN), defined by EPMA. Fig. S5. Elemental distribution determined on a polished thin section of a serpentinized peridotite from an outcrop close to Mountain CEI location, defined by EPMA. Fig. S6. Elemental distribution determined on a polished thin section of a serpentinized peridotite from an outcrop close to Lowland BES location, defined by EPMA. Table S2. Water content, total carbon content and pH of the soils sampled. Table S3. Elemental geochemical composition of the lateritic/serpentine soils from 10 locations of the SEP, analyzed by XRF majors (%) and traces (ppm). Fig. S7. Bulk elemental correlations in the serpentine soils from the SEP for Fe2O3, Mn, Co, Ni, Cr, MgO, Al2O3, and SiO2, all of them analyzed by XRF. Table S3. Linear correlations and probabilities associated for the Fe2O3, Mn, Co, Ni, Cr, MgO, Al2O3, and SiO2, found in serpentine soils from the SEP, all of them analyzed by XRF. Fig. S8. Principal components analysis (PCA) for the geochemistry of the serpentine soils and the score of each variable within each component. Fig. S9. Altitude correlation of the concentration of Fe (a), Mn (b), Ni (c) and Co (d) in the mountain serpentine soils and of Mn (e), Cr (f) and Co (g) in the inner ophiolite lowland serpentine soils. Fig. S10. Rarefac [file 12932_2022_79_MOESM1_ESM.pdf]

Supporting Information for

Geochemistry and microbiology of tropical serpentine soils in the Santa Elena Ophiolite, a landscape-biogeographical approach

Agustín F. Solano-Arguedas[1,2], Christopher Boothman[1], Laura Newsome[1,3], Richard A.D. Pattrick[1], Daniel Arguedas-Quesada[4], Clare H. Robinson[1] and Jonathan R. Lloyd[1]

[1] Williamson Research Centre, Department of Earth and Environmental Sciences, School of Natural Sciences, University of Manchester, Manchester, M13 9PL, United Kingdom.

[2] Forest Resources Unit (Reforesta), Engineering Research Institute (INII), Universidad de Costa Rica, San José, 11501-2260, Costa Rica. *Current Address.

[3] Camborne School of Mines and Environment and Sustainability Institute, University of Exeter, Penryn, Cornwall, TR10 9FE, United Kingdom. *Current Address

[4] Sociedad Civil Pro Ambiente Verdiazul CR, Playa Junquillal de Santa Cruz, Guanacaste, 50303, Costa Rica.

Contents of this file

Figures S1 to S13
Tables S1 to S4

Introduction

The following supporting information comprises additional figures, tables with geochemical data and complementary statistical analyses to support main discussion. For ease of reading, figures and tables are presented as they were mentioned in the main text. This document includes detailed maps of the sampling locations in the Santa Elena Peninsula (SEP) of Costa Rica, tables and figures based on X-ray fluorescence spectroscopy (XRF) and X-ray diffraction spectroscopy (XRD) data of rocks and soils collected, elemental distribution of polished thin sections of rocks analyzed with electron probe micro analysis (EPMA) and supplementary information of fungal and prokaryotic sequencing analyses.

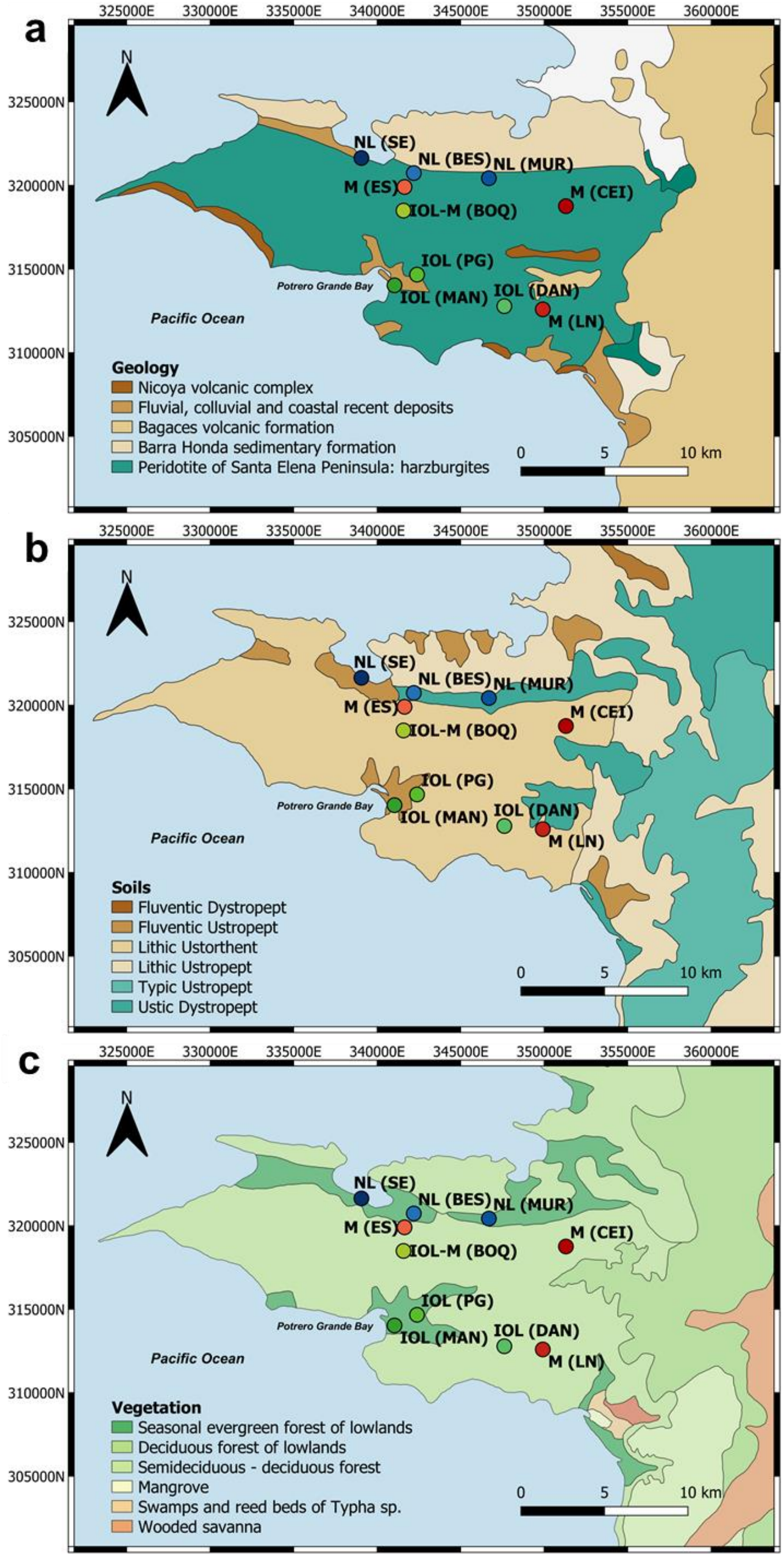

Fig. S1. Geographical distribution of the serpentine soils sampled in 10 locations of the SEP within the maps of the geology (a), soil taxonomy (b) and vegetation (c), only the relevant colors are labelled. Color code for locations is based on the Geochemical-Landscape classification (Fig. 5, 9a): red-to-orange colors assigned to mountain (M) landscapes, green colors to inner ophiolite lowlands (IOL) and light-to-dark blue colors to north lowlands (NL). Maps adapted, respectively, from Medina [1–3].

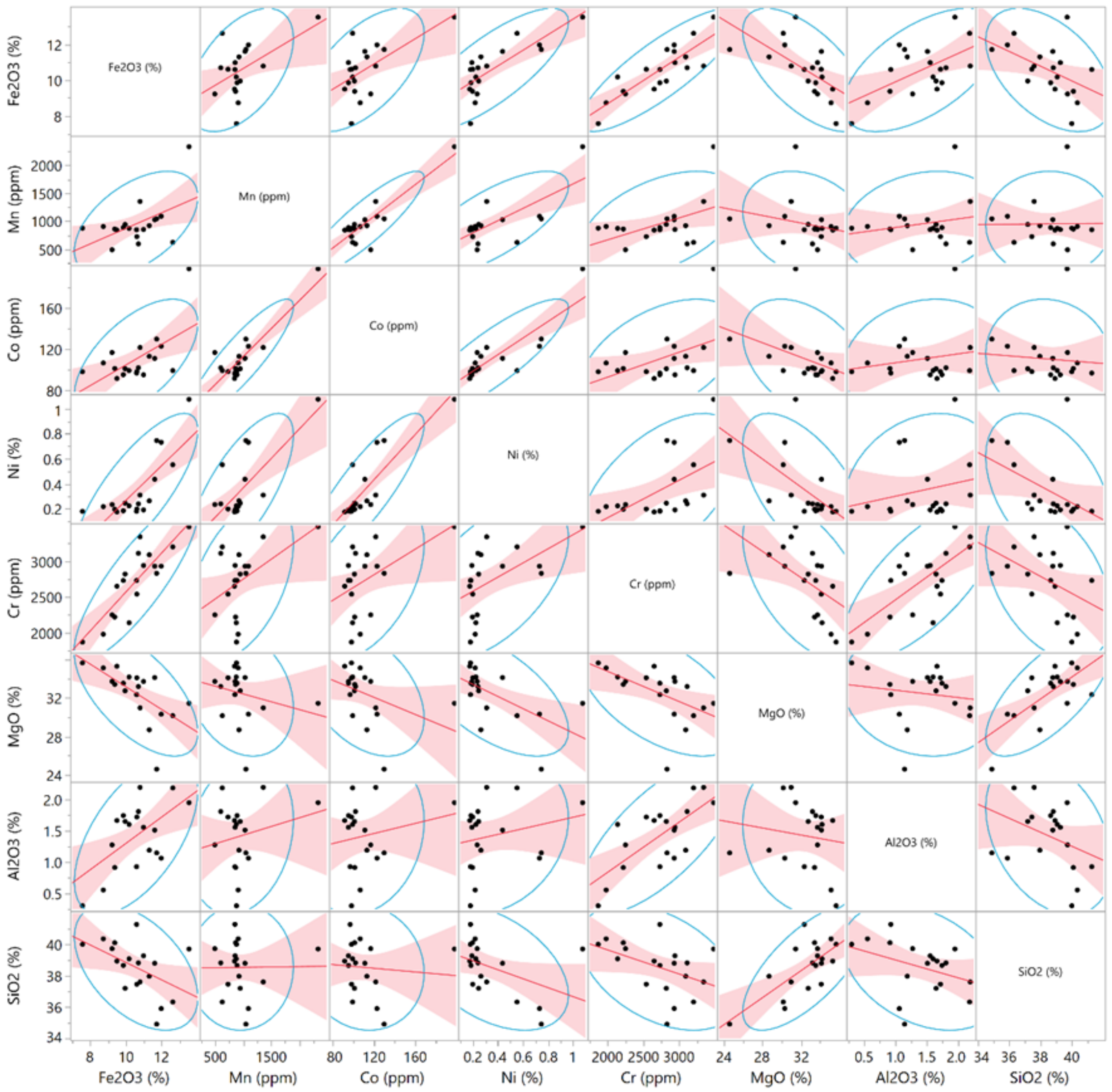

Fig. S2. Bulk elemental correlations in serpentinized rocks from the SEP for Fe_2O_3, Mn, Co, Ni, Cr, MgO, Al_2O_3, and SiO_2, all of them analyzed by XRF. All the rocks samples collected were considered for the analysis (α=95% for density ellipses and confidence curves). Correlations and probabilities associated are shown in Table S1

Table S1. Linear correlations and probabilities associated for Fe_2O_3, Mn, Co, Ni, Cr, MgO, Al_2O_3, and SiO_2 found in serpentinized rocks from the SEP, all of them analyzed by XRF. [a]

**Corr.**[b]	**Fe_2O_3**	**Mn**	**Co**	**Ni**	**Cr**	**MgO**	**Al_2O_3**	**SiO_2**
Fe_2O_3	1.0000	0.5112	0.5959	0.7790	0.8428	-0.6234	0.5784	-0.4802
Mn	0.5112	1.0000	0.8765	0.7175	0.4670	-0.2330	0.2066	0.0113
Co	0.5959	0.8765	1.0000	0.8530	0.4655	-0.3960	0.1864	-0.0853
Ni	0.7790	0.7175	0.8530	1.0000	0.5448	-0.5949	0.2235	-0.4386
Cr	0.8428	0.4670	0.4655	0.5448	1.0000	-0.5367	0.7139	-0.4280
MgO	-0.6234	-0.2330	-0.3960	-0.5949	-0.5367	1.0000	-0.1466	0.7012

Corr.[b]	Fe_2O_3	Mn	Co	Ni	Cr	MgO	Al_2O_3	SiO_2
Al_2O_3	0.5784	0.2066	0.1864	0.2235	0.7139	-0.1466	1.0000	-0.3575
SiO_2	-0.4802	0.0113	-0.0853	-0.4386	-0.4280	0.7012	-0.3575	1.0000
**Prob.[c]**	**Fe_2O_3**	**Mn**	**Co**	**Ni**	**Cr**	**MgO**	**Al_2O_3**	**SiO_2**
Fe_2O_3	<.0001	0.0253	0.0071	<.0001	<.0001	0.0043	0.0095	0.0375
Mn	0.0253	<.0001	<.0001	0.0005	0.0438	0.3372	0.3961	0.9634
Co	0.0071	<.0001	<.0001	<.0001	0.0446	0.0933	0.4448	0.7284
Ni	<.0001	0.0005	<.0001	<.0001	0.0159	0.0072	0.3576	0.0603
Cr	<.0001	0.0438	0.0446	0.0159	<.0001	0.0178	0.0006	0.0675
MgO	0.0043	0.3372	0.0933	0.0072	0.0178	<.0001	0.5494	0.0008
Al_2O_3	0.0095	0.3961	0.4448	0.3576	0.0006	0.5494	<.0001	0.1329
SiO_2	0.0375	0.9634	0.7284	0.0603	0.0675	0.0008	0.1329	<.0001

[a] All the rocks collected were considered for the analysis. [b] Blue color indicates the positive correlations and red the negative ones. [c] Colored numbers indicate $p<0.05$.

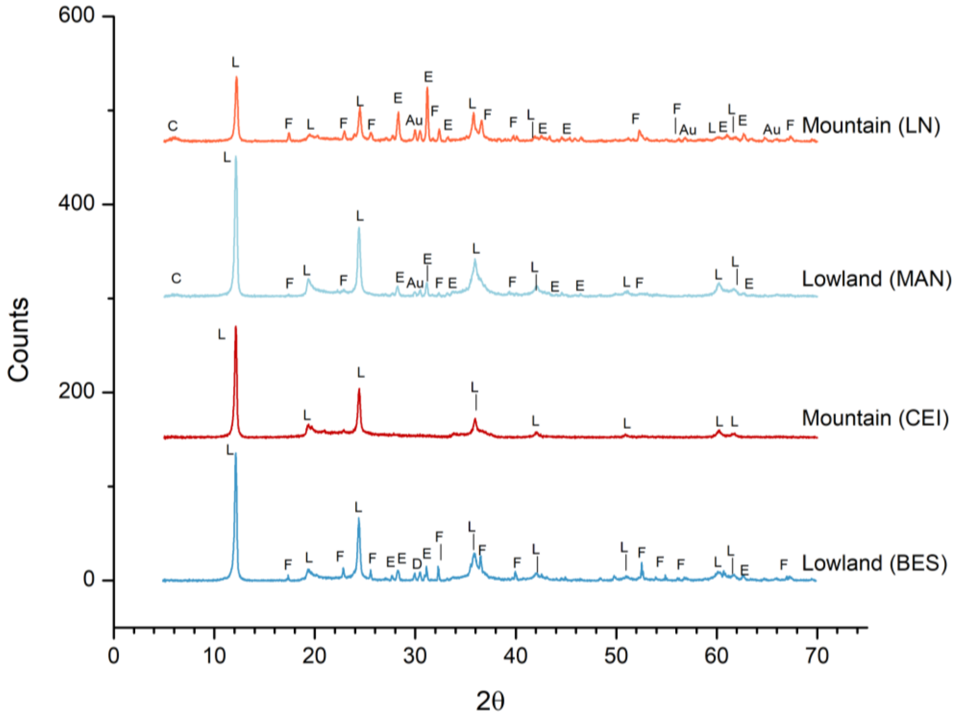

Fig. S3. Bulk mineralogy of rock clasts from the soils (10 cm depth) of the SEP. Main minerals found with XRD were: lizardite (L), enstatite (E), forsterite (F), augite (Au), diopside (D) and clinochlore (C). The samples shown are representative of all the rocks collected in both Mountain and Lowland areas (LN and MAN respectively) and from the superficial outcrops close to sampling locations (CEI and BES)

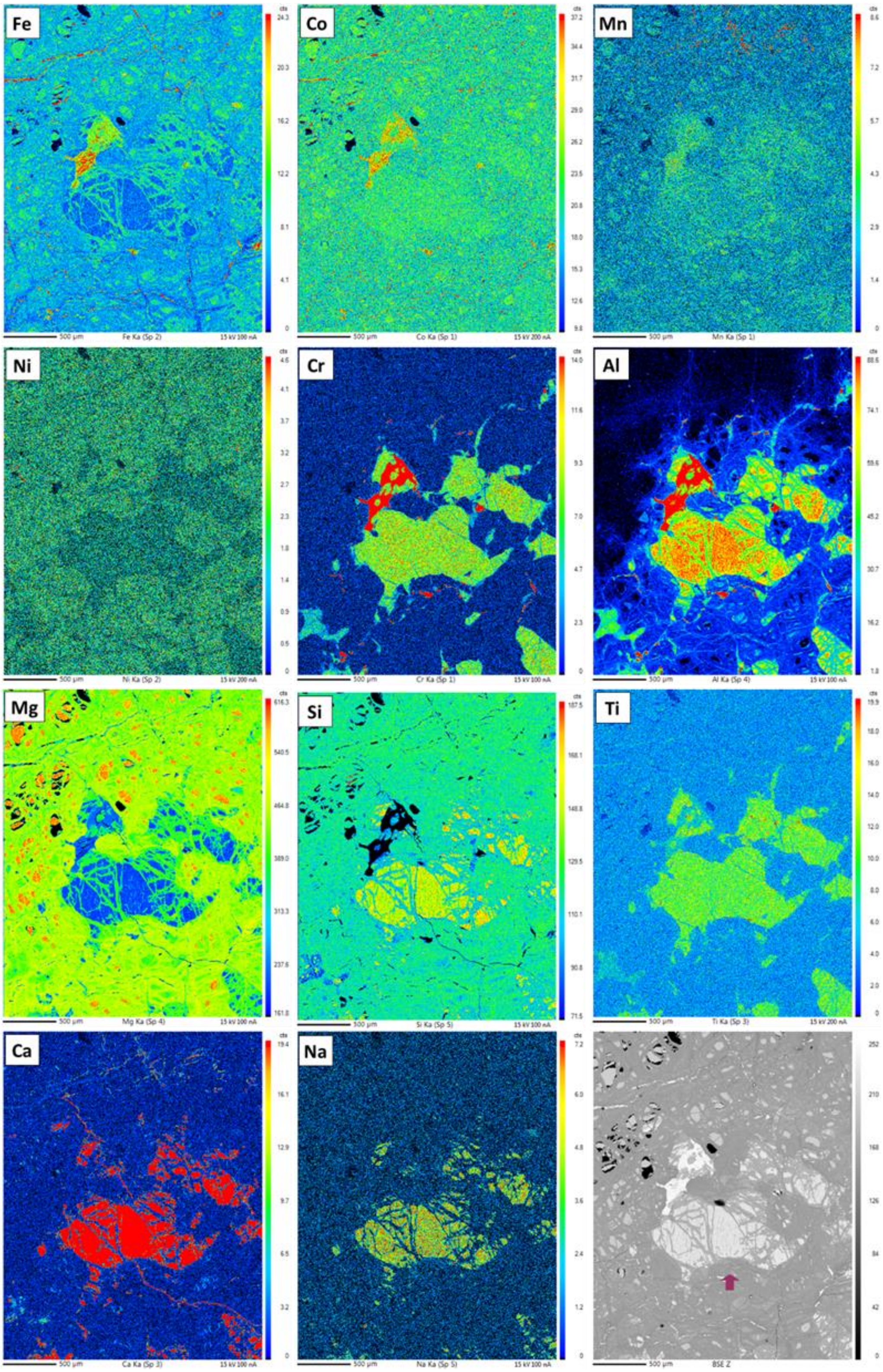

Fig. S4. Elemental distribution determined on a polished thin section of a serpentinite clast from 10 cm depth (Lowland site MAN), defined by EPMA. The area includes that covered by Fig. 2b (rotated 90° clockwise). Count intensity color scale (right of each micrograph) decreases downwards; scale 500 µm

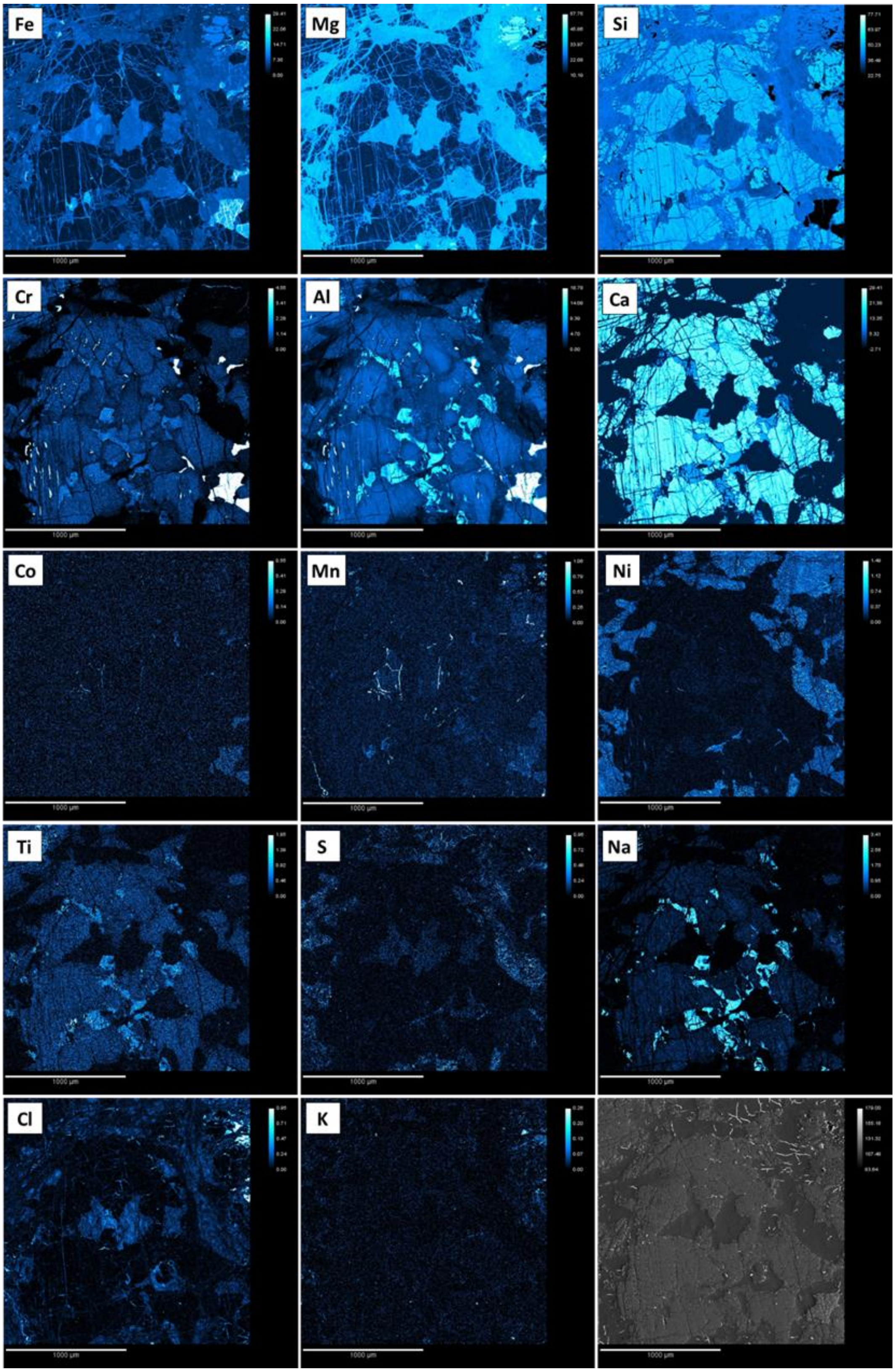

Fig. S5. Elemental distribution determined on a polished thin section of a serpentinized peridotite from an outcrop close to Mountain CEI location, defined by EPMA. The area includes that covered by Fig. 2c (rotated 90° counterclockwise). Count intensity color scale (right of each micrograph) decreases downwards; scale 1000 µm

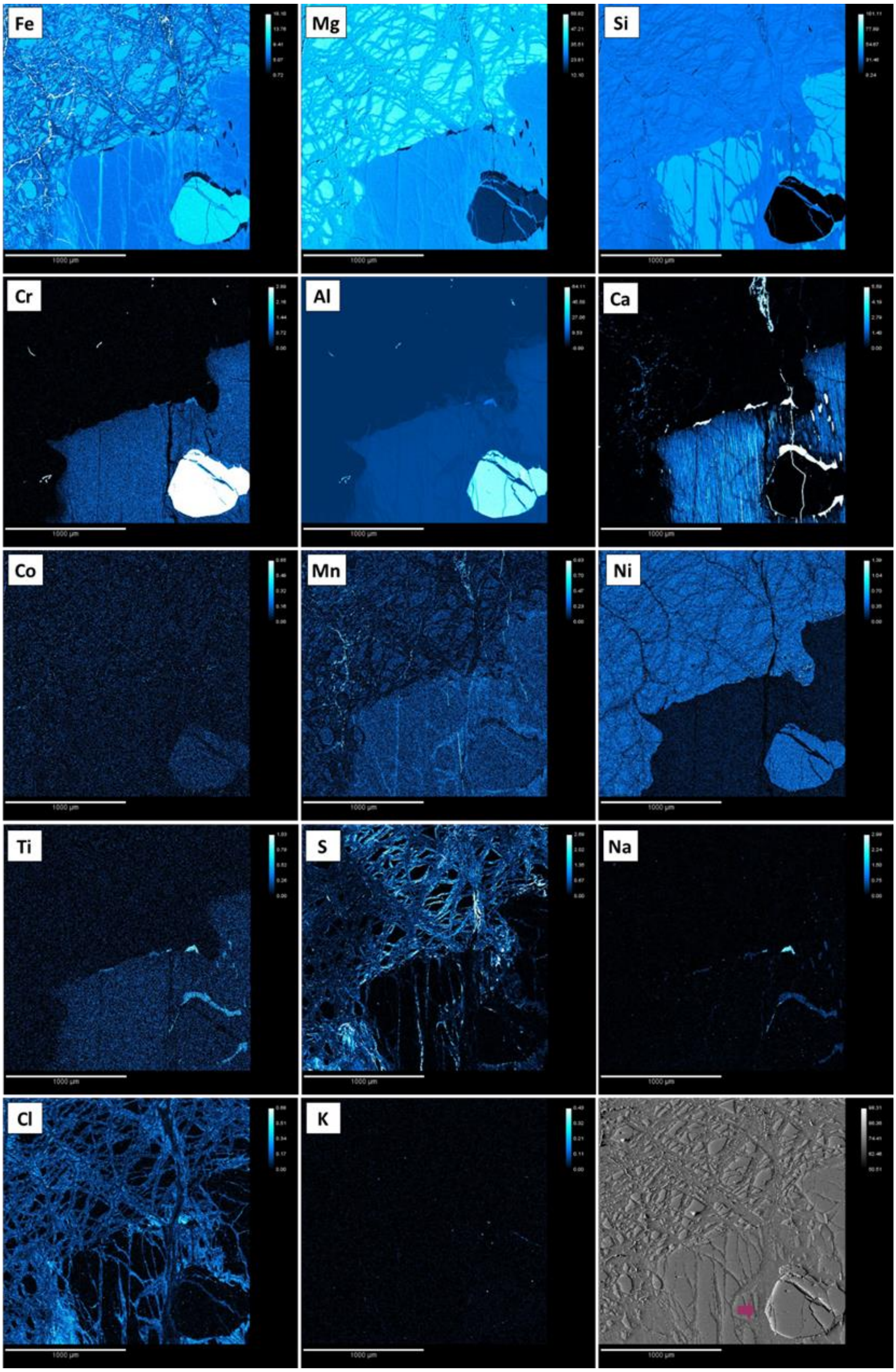

Fig. S6. Elemental distribution determined on a polished thin section of a serpentinized peridotite from an outcrop close to Lowland BES location, defined by EPMA. The area includes that covered by Fig. 2d (rotated 90° clockwise). Count intensity color scale (right of each micrograph) decreases downwards; scale 1000 µm

Table S2. Water content, total carbon content and pH of the soils sampled. [a]

Location	Water (wt%)	Total carbon (wt%) [b]	pH [c]
Mountain (CEI)	27.1 ± 3.7	12.1 ± 1.3	6.94 ± 0.18
Mountain (LN)	26.1 ± 5.9	11.2 ± 0.8	7.21 ± 0.06
Mountain (PG)	20.8 ± 1.3	14.8 ± 0.2	6.95 ± 0.10
Mountain (ES)	26.3 ± 0.9	12.4 ± 0.1	7.15 ± 0.03
Mountain (BOQ)	19.4 ± 2.5	16.2 ± 1.2	6.85 ± 0.50
Lowland (DAN)	11.9 ± 3.2	15.1 ± 0.4	7.35 ± 0.21
Lowland (MAN)	15.4 ± 2.3	10.2 ± 0.5	7.49 ± 0.19
Lowland (BES)	17.2 ± 0.5	12.8 ± 0.2	6.47 ± 0.14
Lowland (MUR)	19.5 ± 0.8	11.9 ± 0.3	6.35 ± 0.07
Lowland (SE)	8.6 ± 3.7	11.1 ± 1.5	6.83 ± 0.07

[a] Results are shown as an average of the 3 replicates collected from every location and their standard deviation. [b] Total carbon content was calculated for dry weight. [c] pH was measured in fresh samples.

The water content of the soils was higher in the mountain locations (23.9 ± 4.3 %) than in the lowland ones (14.5 ± 4.5 %) ($F=33.52$, $p<0.0001$). There was no difference between the landscapes in terms of the total carbon content of the samples (Mountain: 13.4 ± 2.1 wt%, Lowland: 12.2 ± 1.9 wt%, $F=2.57$, $p=0.1204$), and pH was circumneutral for all the locations (Mountain: 7.01 ± 0.25, Lowland: 6.90 ± 0.49; $Z=0.83$, $p=0.4065$) (Table S2).

Table S3. Elemental geochemical composition of the lateritic/serpentine soils from 10 locations of the SEP, analyzed by XRF majors (%) and traces (ppm).

Site	Fe_2O_3 (%) [a]	MgO (%)	SiO_2 (%)	Al_2O_3 (%)	CaO (%)	TiO_2 (%)	Na_2O (%)	MnO (%)	Ni (%)	Cr (%)
Mountain (CEI)	41.6 ± 9.5	9.0 ± 6.4	23.8 ± 7.3	4.4 ± 2.5	0.22 ± 0.05	0.17 ± 0.09	0.05 ± 0.01	0.51 ± 0.11	1.26 ± 0.14	0.92 ± 0.28
Mountain (LN)	27.2 ± 5.0	16.7 ± 2.8	32.9 ± 2.9	2.5 ± 0.3	0.28 ± 0.08	0.09 ± 0.01	0.02 ± 0.04	0.34 ± 0.07	1.18 ± 0.19	0.75 ± 0.08
Mountain (PG)	25.7 ± 6.2	13.3 ± 4.1	31.0 ± 3.8	5.6 ± 1.6	0.45 ± 0.16	0.20 ± 0.09	0.02 ± 0.03	0.35 ± 0.05	0.68 ± 0.08	0.68 ± 0.17
Mountain (ES)	27.0 ± 2.0	14.8 ± 1.9	33.7 ± 1.5	5.0 ± 0.9	0.14 ± 0.02	0.12 ± 0.04	0.04 ± 0.01	0.32 ± 0.05	0.84 ± 0.11	0.76 ± 0.04
Mountain (BOQ)	17.7 ± 3.2	15.0 ± 11.5	35.8 ± 2.7	13.6 ± 10.4	0.47 ± 0.22	0.57 ± 0.48	0.06 ± 0.01	0.24 ± 0.03	0.41 ± 0.22	0.29 ± 0.04
Lowland (DAN)	18.7 ± 3.5	22.1 ± 5.3	36.7 ± 1.6	6.6 ± 2.6	0.95 ± 0.30	0.25 ± 0.10	0.13± 0.05	0.26 ± 0.04	0.49 ± 0.05	0.59 ± 0.16
Lowland (MAN)	18.4 ± 0.4	15.3 ± 0.5	39.2 ± 1.0	8.5 ± 0.3	2.35 ± 0.22	0.46 ± 0.10	0.22 ± 0.04	0.48 ± 0.06	0.55 ± 0.01	0.73 ± 0.15
Lowland (BES)	24.9 ± 0.5	4.4 ± 1.4	32.9 ± 1.1	16.1 ± 1.5	1.64 ± 0.14	0.90 ± 0.03	0.14± 0.03	0.46 ± 0.01	0.38 ± 0.05	0.68 ± 0.01
Lowland (MUR)	19.1 ± 0.8	3.3 ± 0.6	35.8 ± 0.6	21.5 ± 1.5	1.70 ± 0.35	1.00 ± 0.11	0.13 ± 0.03	0.30 ± 0.01	0.24 ± 0.02	0.28 ± 0.07
Lowland (SE)	16.4± 0.5	10.4 ± 0.5	41.6 ± 0.1	13.8 ± 0.3	3.85 ± 0.19	0.77 ± 0.03	0.89 ± 0.03	0.272 ± 0.003	0.18 ± 0.01	0.185 ± 0.003

[a] All soils are high in iron oxides

Continuation. Table S3. Elemental geochemical composition of the lateritic/serpentine soils from 10 locations of the SEP, analyzed by XRF majors (%) and traces (ppm).

Site	Co (ppm)	V (ppm)	Zn (ppm)	Cu (ppm)	Ba (ppm)	Te (ppm)	Sc (ppm)	Sr (ppm)	Zr (ppm)	I (ppm)
Mountain (CEI)	359 ± 42	135 ± 53	131 ± 28	83 ± 27	74 ± 25	61 ± 2	33 ± 10	19 ± 4	22 ± 14	36 ± 8
Mountain (LN)	289 ± 60	116 ± 8	107 ± 5	56 ± 7	59 ± 17	59 ± 3	23 ± 1	17 ± 11	7 ± 1	11 ± 10
Mountain (PG)	219 ± 41	120 ± 39	79 ± 14	68 ± 14	60 ± 7	55 ± 3	28 ± 7	15 ± 4	16 ± 7	13 ± 4
Mountain (ES)	253 ± 31	98 ± 9	70 ± 8	59 ± 27	34 ± 10	55 ± 3	32 ± 5	7 ± 1	4 ± 1	18 ± 3
Mountain (BOQ)	125 ± 48	181 ± 104	48 ± 14	74 ± 29	46 ± 22	52 ± 2	32 ± 16	14 ± 15	35 ± 33	12 ± 8
Lowland (DAN)	163 ± 20	117 ± 39	85 ± 17	55 ± 19	102 ± 35	51 ± 2	19 ± 5	30 ± 23	23 ± 12	12 2
Lowland (MAN)	250 ± 22	160 ± 8	126 ± 25	64 ± 13	95 ± 12	52 ± 3	25 ± 1	21 ± 2	25 ± 1	0
Lowland (BES)	197 ± 4	254 ± 25	118 ± 9	110 ± 9	175 ± 11	55 ± 1	39 ± 3	43 ± 7	74 ± 10	26 ± 2
Lowland (MUR)	102 ± 7	290 ± 6	58 ± 9	106 ± 7	159 ± 7	53 ± 2	44 ± 2	42 ± 10	77 ± 5	14 ± 1
Lowland (SE)	94 ± 3	196 ± 4	86 ± 3	103 ± 7	59 ± 2	52 ± 1	29.7 ± 0.1	77 ± 3	53 ± 1	8 ± 2

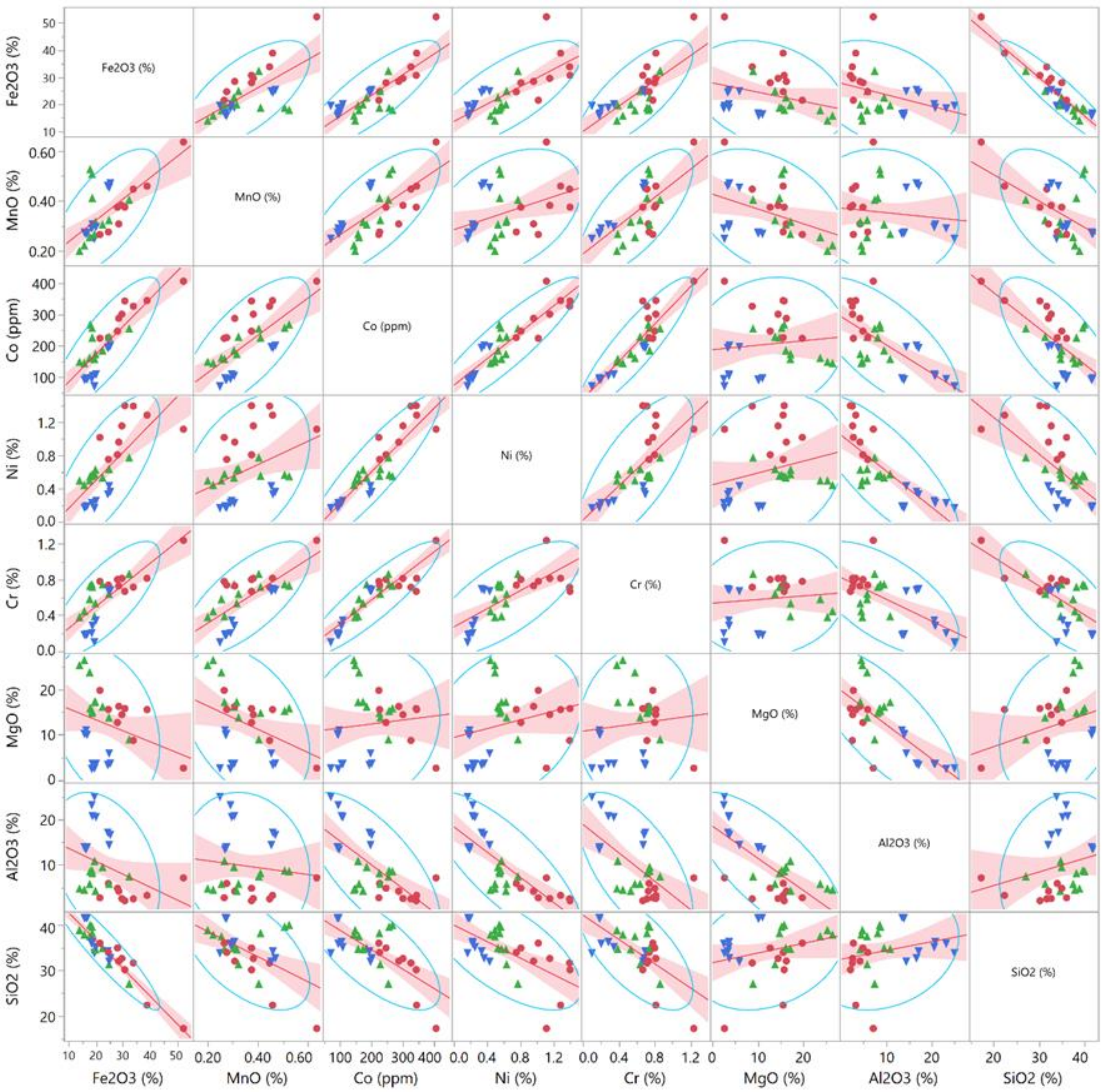

Fig. S7. Bulk elemental correlations in the serpentine soils from the SEP for Fe_2O_3, Mn, Co, Ni, Cr, MgO, Al_2O_3, and SiO_2, all of them analyzed by XRF. All the soil samples collected were considered for the analysis (α=95% for density ellipses and confidence curves). Correlations and probabilities are shown in Table S4. Markers correspond to clusters of Fig. 5b, 9a: mountain soils (●), inner ophiolite lowland soils (▲) and north lowland soils (▼)

Table S4. Linear correlations and probabilities associated for the Fe_2O_3, Mn, Co, Ni, Cr, MgO, Al_2O_3, and SiO_2, found in serpentine soils from the SEP, all of them analyzed by XRF.

Corr.[a]	Fe_2O_3	Mn	Co	Ni	Cr	MgO	Al_2O_3	SiO_2
Fe_2O_3	1.0000	0.6661	0.8229	0.7339	0.7505	-0.2871	-0.3478	-0.9319
Mn	0.6661	1.0000	0.7099	0.3940	0.7096	-0.3890	-0.1192	-0.5399
Co	0.8229	0.7099	1.0000	0.8986	0.8951	0.1121	-0.6862	-0.6993
Ni	0.7339	0.3940	0.8986	1.0000	0.7201	0.2619	-0.7775	-0.6340
Cr	0.7505	0.7096	0.8951	0.7201	1.0000	0.1056	-0.6167	-0.6555
MgO	-0.2871	-0.3890	0.1121	0.2619	0.1056	1.0000	-0.7302	0.2731
Al_2O_3	-0.3478	-0.1192	-0.6862	-0.7775	-0.6167	-0.7302	1.0000	0.2409
SiO_2	-0.9319	-0.5399	-0.6993	-0.6340	-0.6555	0.2731	0.2409	1.0000
**Prob.[b]**	**Fe_2O_3**	**Mn**	**Co**	**Ni**	**Cr**	**MgO**	**Al_2O_3**	**SiO_2**
Fe_2O_3	<.0001	<.0001	<.0001	<.0001	<.0001	0.1240	0.0596	<.0001
Mn	<.0001	<.0001	<.0001	0.0312	<.0001	0.0336	0.5303	0.0021
Co	<.0001	<.0001	<.0001	<.0001	<.0001	0.5553	<.0001	<.0001
Ni	<.0001	0.0312	<.0001	<.0001	<.0001	0.1621	<.0001	0.0002
Cr	<.0001	<.0001	<.0001	<.0001	<.0001	0.5786	0.0003	<.0001
MgO	0.1240	0.0336	0.5553	0.1621	0.5786	<.0001	<.0001	0.1442
Al_2O_3	0.0596	0.5303	<.0001	<.0001	0.0003	<.0001	<.0001	0.1998
SiO_2	<.0001	0.0021	<.0001	0.0002	<.0001	0.1442	0.1998	<.0001

[a] Blue color indicates the positive correlations and red the negative ones. [b] Colored numbers indicate $p<0.05$.

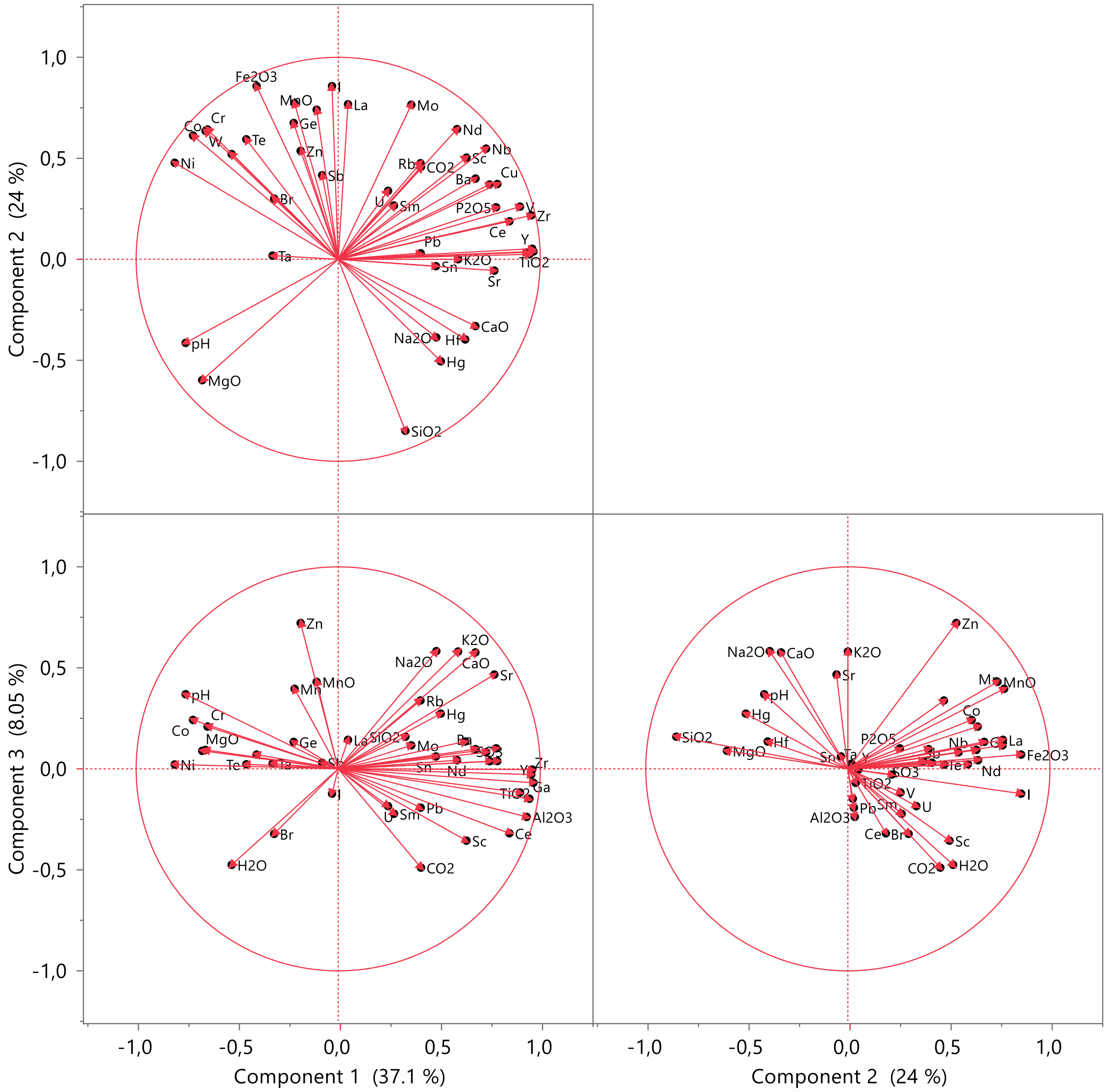

Fig. S8. Principal components analysis (PCA) for the geochemistry of the serpentine soils and the score of each variable within each component. Three main components explained ~69% of the variance within the samples according to PCA: PC1 (37.1%), PC2 (24%) and PC3 (8.05%)

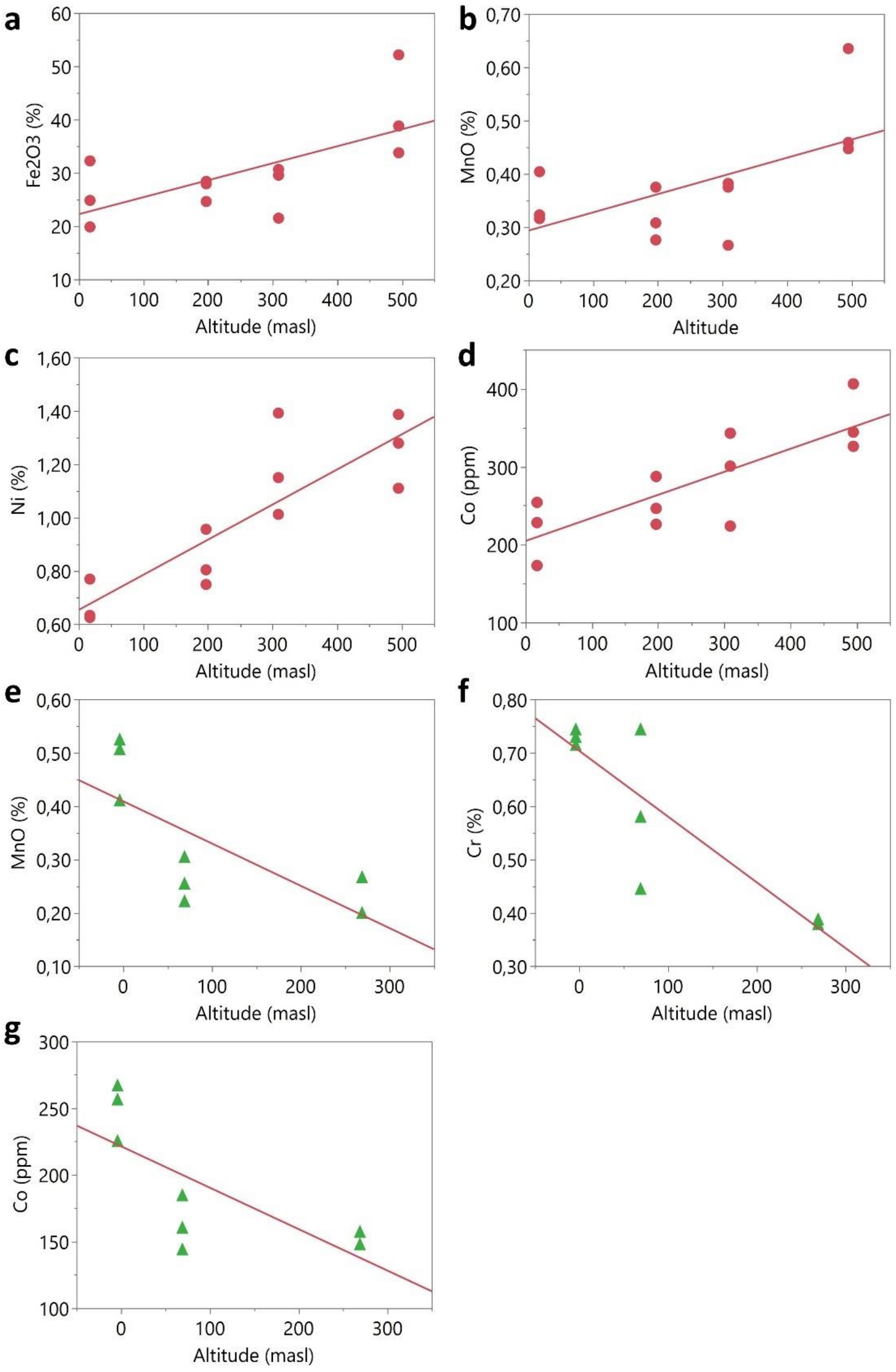

Fig. S9. Altitude correlation of the concentration of Fe (a), Mn (b), Ni (c) and Co (d) in the mountain serpentine soils and of Mn (e), Cr (f) and Co (g) in the inner ophiolite lowland serpentine soils. Masl: meters above sea level

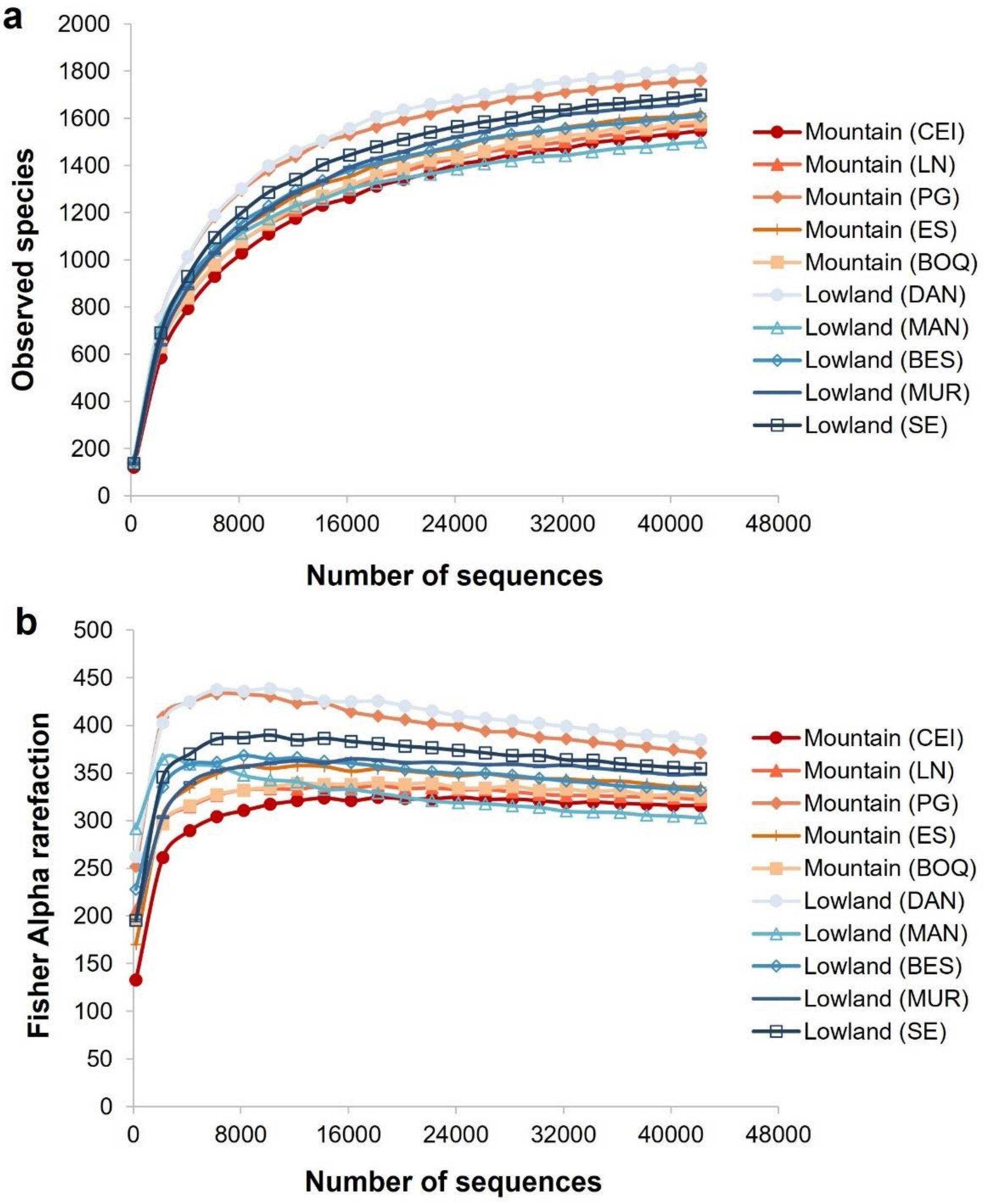

Fig. S10. Rarefaction curves for the prokaryotic communities in the serpentine soils overall the locations sampled in the SEP per observed species (a) and Fisher Alpha (b) after sequencing the V4 region of 16S rRNA

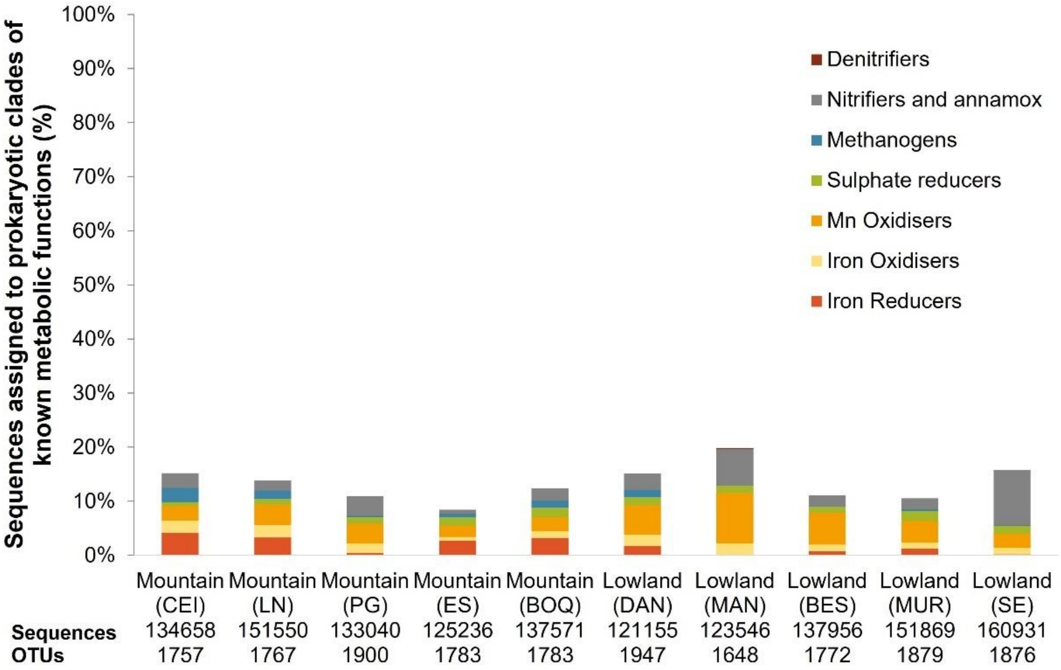

Fig. S11. Abundance of sequences per prokaryotic metabolic function assigned to phylogenetic clades after sequencing the V4 region of 16S rRNA in the serpentine soils overall the locations from the SEP

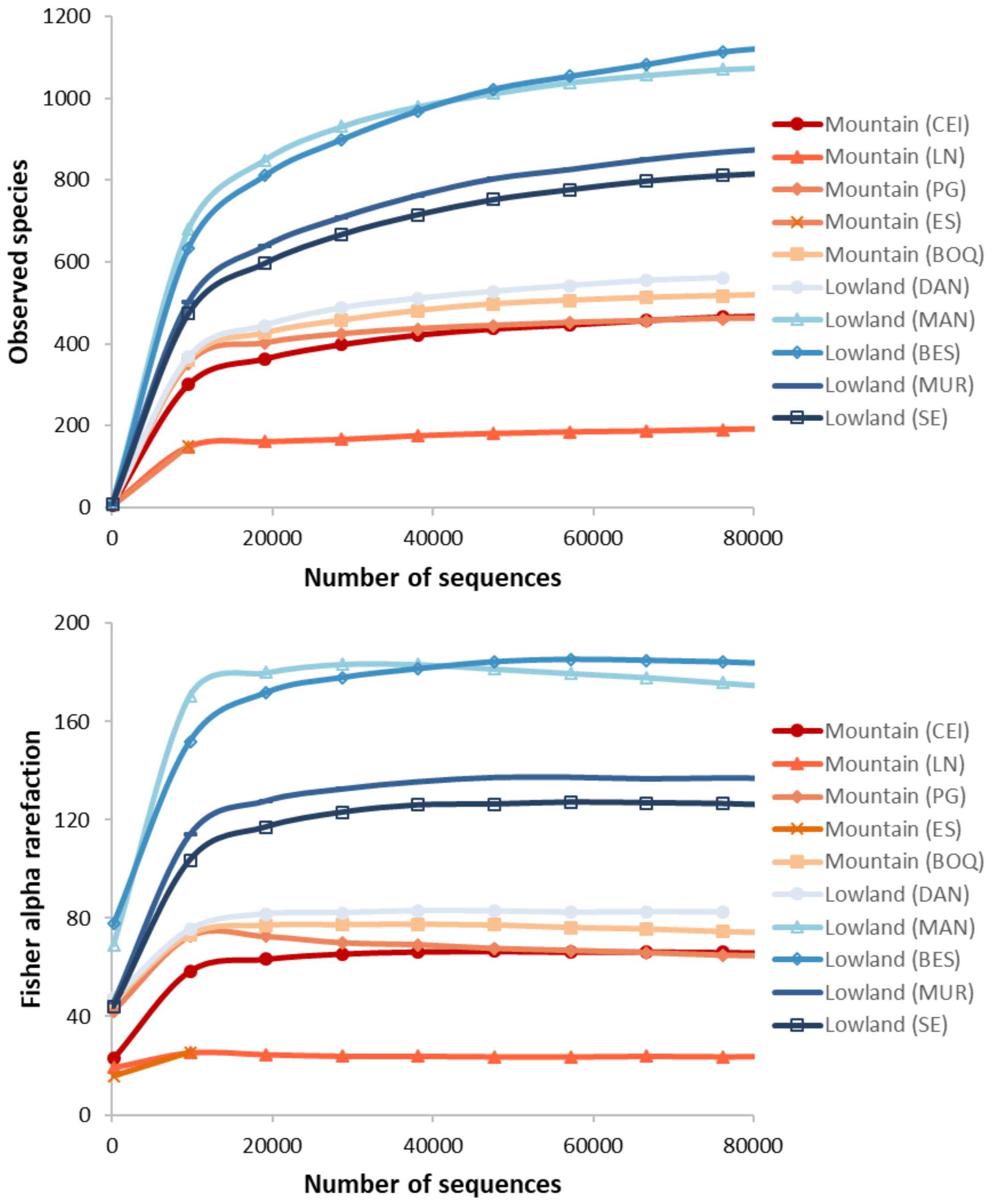

Fig. S12. Rarefaction curves for the fungal communities in the serpentine soils overall the locations sampled in the SEP per observed species (top) and Fisher Alpha (bottom) after sequencing the ITS2 region of nuclear ribosomal DNA

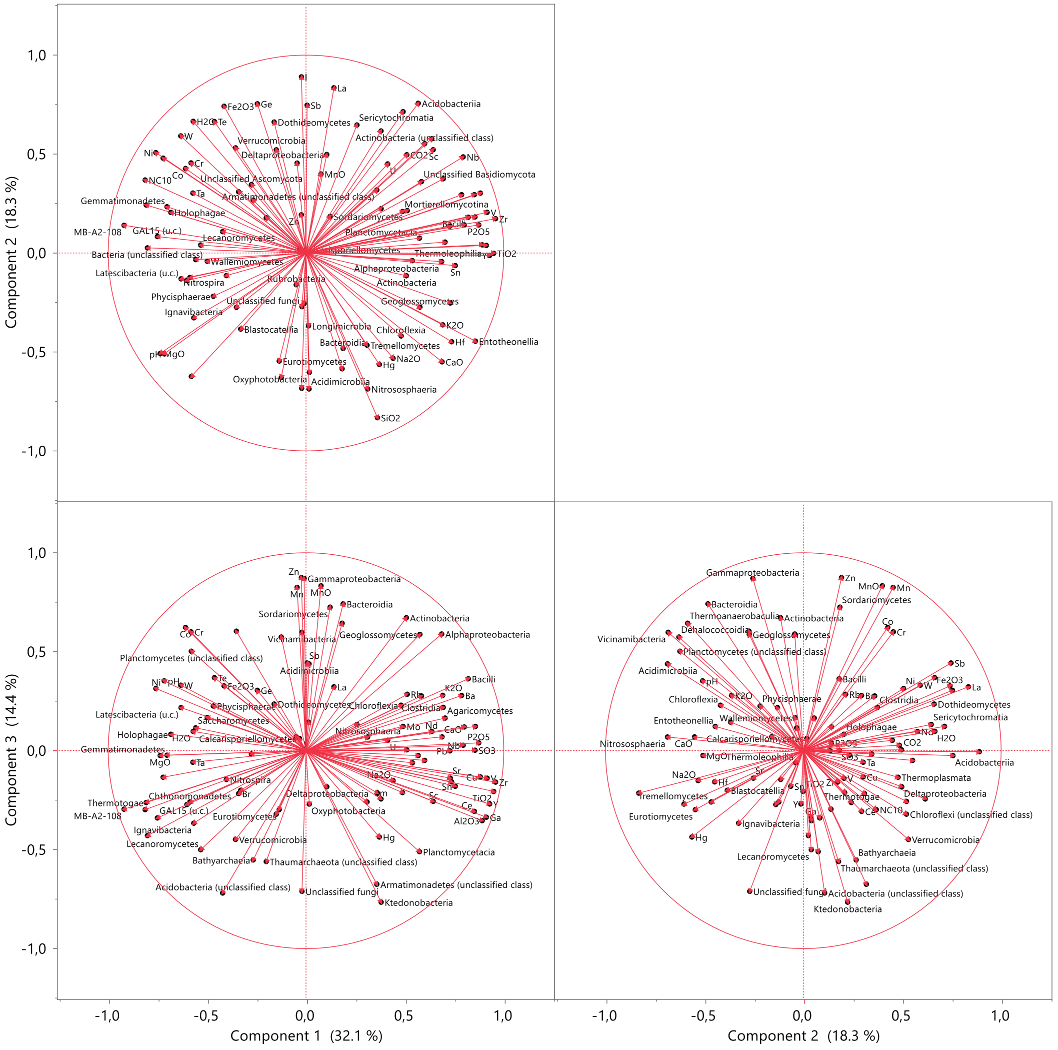

Fig. S13. Principal components analysis (PCA) for microbial-geochemical associations in the serpentine soils from the Santa Elena and the score of each variable within each component. Three main components explained ~64% of the variance within the samples: PC1 (32.1%), PC2 (18.3%) and PC3 (14.4%)

References

1. Medina W (1999) Geología: Hoja Liberia - Area de Conservación Guanacaste. In: Sist. Inf. Geográfica - Área Conserv. Guanacaste. http://www.investigadoresacg.org/IMAGES/MAPFILES/maps/physical_biological/geologia_acg.jpg. Accessed 11 Dec 2016
2. Medina W (1999) Suelos: Hoja Liberia - Area de Conservación Guanacaste. In: Sist. Inf. Geográfica - Área Conserv. Guanacaste. http://www.investigadoresacg.org/IMAGES/MAPFILES/maps/physical_biological/suelos_acg.jpg. Accessed 11 Dec 2016
3. Medina W (1999) Tipos de vegetación: Hoja Liberia - Area de Conservación Guanacaste. In: Sist. Inf. Geográfica - Área Conserv. Guanacaste. http://www.investigadoresacg.org/IMAGES/MAPFILES/maps/physical_biological/vegetacion_acg.jpg. Accessed 11 Dec 2016
